# Soil pH mediates the impact of pesticides on bacterial communities, diversity, and abundance

**DOI:** 10.3389/fmicb.2025.1670425

**Published:** 2026-01-16

**Authors:** Ema Némethová, Milan Řezáč, Milan Gryndler, Oushadee A. J. Abeyawardana, Veronika Řezáčová

**Affiliations:** 1 Czech Agrifood Research Center, Prague, Czechia; 2 Faculty of Science, J. E. Purkyně University in Ústí nad Labem, České Mládeže, Ústí nad Labem, Czechia

**Keywords:** herbicides, fungicides, bacteria, diversity, real-time PCR, rhizobia

## Abstract

**Introduction:**

Pesticides are widely used in agriculture, yet their non-target effects on soil microbial communities remain poorly understood. This study investigates the short-term impact of five herbicides and three fungicides used for the protection of legumes on the composition and diversity of soil bacteria, with special focus on rhizobia.

**Methods:**

Using three distinct soils from ecologically maintained fields, we assessed changes in bacterial communities and total bacterial abundance in response to different active substances under controlled conditions, 2 weeks after pesticide application. Bacterial diversity was analyzed by amplifying and sequencing the V4 region of the 16S rRNA gene via Illumina paired-end amplicon sequencing. Real-time PCR was used to assess total abundance of bacteria.

**Results:**

Our results show that pesticide effects on bacteria are highly context-dependent, influenced significantly by soil and pH. Significant changes in bacterial diversity were detected only in one soil, whereas in another soil no significant differences among individual pesticides and the untreated control were found. In this soil, particularly the CORUM and pendimethalin-based products, Stomp 400 SC and Sharpen 40 SC, consistently reduced bacterial diversity, while some pesticides had a neutral effect. Rhizobial diversity remained largely unaffected, suggesting greater resilience compared to general bacterial communities. Regarding bacterial community composition, only some pesticides significantly affected bacterial community structure in each soil, and the pesticides showing this effect differed among soils. Redundancy analysis revealed that pH was a stronger driver of bacterial community structure than soil type or pesticide identity, explaining over 60% of community variability.

**Conclusion:**

These findings highlight the complex interactions between pesticides, soil characteristics, and microbial communities. Our results support considering soil pH when selecting pesticides to support sustainable soil management and minimize ecological disruption.

## Introduction

The rapid growth of the global population has driven the need for increased agricultural productivity, a goal that has largely been achieved through the use of chemical fertilizers and pesticides. These chemicals play a crucial role in protecting crops from pests and diseases, thereby ensuring higher crop yields. However, the widespread use of pesticides has raised significant concerns regarding their potential risks to environmental sustainability and ecological stability. One of the most pressing issues is the detrimental impact of pesticides on soil microorganisms, including those essential for plant growth, biodiversity, and the functioning of food webs (Santos and Flores, 1995; Pagano et al., 2023; Wan et al., 2025).

Soil microorganisms are involved in numerous vital ecological processes, such as nutrient cycling, soil structure formation, and the natural protection of plants against pathogens (Hartmann et al., 2006; Nikitin et al., 2022; Řezáčová et al., 2021a, 2023). Furthermore, many microorganisms have the ability to degrade toxic substances, including pesticides, and contribute to the resilience of ecosystems (Lenoir et al., 2016; El-Sayed et al., 2018; Chia et al., 2024). However, excessive pesticide application can disrupt these beneficial interactions, leading to a reduction in plant protection and overall soil health (Wan et al., 2025; Daisley et al., 2022; Jeyaseelan et al., 2024). To mitigate these negative consequences, it is essential to evaluate the specific impacts of various pesticides on non-target soil microorganisms. This knowledge is critical for developing alternative management strategies that promote environmental sustainability.

Free-living soil bacteria, as well as those in symbiosis with plants, can also promote plant growth by producing growth-stimulating compounds, offering protection against pathogens, or contributing to soil aggregation (Hashem et al., 2019; Řezáčová et al., 2021a; Aioub et al., 2022). Among these, rhizobia, a group of bacteria within the *Rhizobiales* order, facilitate nitrogen uptake by plants and help protect them from disease (Masson-Boivin and Sachs, 2018; Aioub et al., 2022). Rhizobia also influence the composition of the soil microbiome (Lu et al., 2017; Ma et al., 2024), thereby affecting several soil functions, such as the production of biofilms that enhance soil aggregate stability (Qurashi and Sabri, 2012; Lehmann et al., 2017).

In agroecosystems, herbicides are the most commonly used pesticides (Maino et al., 2023). Herbicides, typically applied via foliar sprays, accumulate in the soil, potentially affecting both free-living soil bacteria and microbial symbionts (Fuchs et al., 2023; Wang et al., 2024). Fungicides, on the other hand, are often applied directly to seeds or plant tissues, thereby interacting more directly with these microorganisms. Research has shown that pesticides can inhibit various soil bacteria species (Onwona-Kwakye et al., 2020), interfere with rhizobial symbiosis (Fox et al., 2007; Paniagua-López et al., 2023), and decrease bacterial diversity (Al-Ani et al., 2019; Senabio et al., 2024). These chemicals not only affect bacterial growth but can also impair their beneficial physiological activities that are vital for plant health (Fox et al., 2007; Hussain et al., 2009; Afata et al., 2024). However, there are instances where pesticides exhibit neutral (Khmelevtsova et al., 2023; Daisley et al., 2022) or even stimulatory effects on certain bacterial populations (Sim et al., 2022; Yu et al., 2023). The range of effects is highly variable, influenced by factors such as application method, pesticide concentration, soil type, and the composition of the resident microbial community (Gundi et al., 2005; Akter et al., 2023; Meidl et al., 2024). Importantly, soil pH has emerged as one of the key edaphic drivers shaping microbial community structure, diversity and function (Řezáčová et al., 2019; Xiong et al., 2024; Bradbury et al., 2024). These findings underline that pesticide effects cannot be interpreted in isolation, but must be considered within the context of soil physicochemical properties, especially pH, which may modulate microbial responses to chemical stress. Using soils with differing physicochemical properties, such as pH, texture, and organic matter content, can help capture this natural variability and improve understanding of how soil characteristics mediate microbial responses to pesticides. Additionally, there is a large number of pesticides whose impacts on these crucial bacterial symbionts remain unexplored. Recent meta-analysis by Ni et al. (2025) further showed that increasing pesticide diversity impairs soil microbial functions across field settings.

Given these gaps in knowledge, it is essential to further assess the effects of different pesticides on bacterial biodiversity and to examine how these impacts vary across different soil types. Such investigations will provide valuable insights into the broader ecological consequences of pesticide use and guide the development of more sustainable agricultural practices.

The aim of the study was to evaluate the short-term impact of a range of fungicides and herbicides on the diversity, community composition (relative abundances of different bacterial taxa), and total abundance of bacteria (estimated as the number of 16S rRNA gene copies for Eubacteria and Archaea here). We hypothesized that the effects of these pesticides on bacteria would alter their communities and reduce both diversity and total abundance.

## Materials and methods

### Description of the experiment

The research was based on a pot experiment in which pesticides (3 fungicides and 5 herbicides) were applied to the surface of three different soils. The experiment was a fully factorial design with two factors: (1) pesticide (used pesticide) and (2) soil (used soil). Four biological replicate pots were established per each combination of soil and pesticide, resulting in a total of 108 pots (3 soils × 9 treatments × 4 replicates).

Each treatment included an untreated control for each soil, resulting in 9 treatment levels (8 pesticides + 1 control) per soil.

The soil used in the experiment was sourced from private farmers: (i) VH AGROTON, s.r.o., Velké Hostěrádky (GPS: 49°1′52.43″N, 16°52′20.64″E) (soil 1); (ii) BIOFARMA Sasov, Jihlava (GPS: 49° 22′51.69″N, 15° 36′18.014″E) (soil 2); and (iii) Ing. Přemysl Čech, Zábřeh (GPS: 49°52′59.41″N, 16°51′50.47″E) (soil 3), and had not been treated with pesticides for at least 10 years (Table 1).

**Table 1:** Characteristics of the soils used.

Soil parameter	Soil 1	Soil 2	Soil 3
Available P (mg/kg)	11.25	111.5	101.6
Mg (mg/kg)	410	178.8	114.9
K (mg/kg)	214.0	271.0	280.8
Ca (mg/kg)	7,854	1863	2,198
Hot water extractable P (mg/kg)	3.294	12.37	11.93
NH_4_ (mg/kg)	6.339	10.15	11.18
NO_3_ (mg/kg)	7.949	13.31	13.95
pH_water_	8.2	6.4	7.0

The three soils were analyzed for available nutrients prior to the establishment of the experiment. The soil characteristics as available P, K, Mg, Ca, and N (NO3 and NH4) were measured as in Řezáčová et al. (2021c) in the homogenized dried soil samples. Available P, K, Mg, and Ca were assessed according to the Mehlich III method (Mehlich, 1984) on an Agilant ICP-OES 5110 VDV instrument (Agilent Technologies, Santa Clara, CA, USA). NO3 and NH4 were determined using calcium chloride solution as extractant according to ISO 14255:1998 on an automated chemistry analyser SKALAR (Skalar Analytical B. V., Breda, The Netherlands).

Fresh soil, homogenized by thorough mixing, was added to 1-L pots sterilized with 96% ethanol (Penta, Prague, Czech Republic). The soil was then watered, and the pesticide was evenly applied to the surface.

Finally, the pots were randomly arranged within the area to minimize positional effects.

To assess the impact of pesticides on microbial communities, we selected fungicides and herbicides commonly used for legume protection. Our focus was not only on bacteria as broad taxonomic group but also specifically on rhizobia, a key group of nitrogen-fixing bacteria that form symbiotic relationships with legumes. The tested fungicides were Mirador XTRA, Kuprikol 50, and Captan 80 WG. The selected herbicides were Basagran, CORUM, Stomp 400 SC, Targa Super 5 EC, and Sharpen 40 SC.

Soils, including their microbial communities, were exposed to pesticides at the maximum recommended field dose (as given in Table 2) to visualize the highest pesticide impact that may occur under field conditions. We diluted the pesticides in 0.5 L of water, calculated volume of the solution to be applied corresponding to the maximum recommended dose, and applied it evenly to the soil surface in the experimental pots using a handheld sprayer. Each treatment was applied once at the beginning of the experiment. The soil surface per pot was equal to 169 cm^2^.

**Table 2:** Doses and dilution of individual pesticides applied to soil surface.

Formulation	Manufacturer	Active compound	Dose used [L/ha]	Recommended dose range [L/ha]	Dilution [L/ha]	Recommended dilution [L/ha]
Basagran	BASF AG, Agricultural Products	Bentazone	2	2	200	200–400
CORUM	BASF AG, Agricultural Products	Imazamox, bentazone	1.25	1.25	100	100–400
Sharpen 40 SC	Sharda Worldwide Exports Pvt. Ltd.	Pendimethalin	4.1	4.1	400	400–600
Stomp 400 SC	BASF AG, Agricultural Products	Pendimethalin	4.1	4.1	400	400–600
Targa Super 5 EC	Nissan Chemical Ind. Ltd.	Quizalofop-P-ethyl	2.5	1.5–2.5	200	200–400
Captan 80 WG	Arysta LifeScience S.A.S	Captan	2.1	2.1	500	500
Kuprikol 50	NeraAgro,spol. s r.o.	Copper oxychloride	5	5	200	200
Mirador XTRA	Adama CZ s.r.o.	Azoxystrobin, cyproconazole	1	1	200	200–400

The experiment was conducted in a room with an average temperature of 21.4 °C. Sampling for further analyses was performed after 2 weeks to allow the degradation of DNA from lysed microorganisms in the soil, as microbial DNA typically degrades within hours to a few days in biologically active soils, while only a minor fraction may persist longer when adsorbed to mineral or organic particles (Levy-Booth et al., 2007; Pietramellara et al., 2009; Nielsen et al., 2007). Soil samples were collected from multiple points within the pot at a depth of 0–10 cm using a sterile metal spoon, then combined into a single composite sample and homogenized.

### Sample analyses

After air-drying the soil at room temperature and homogenizing it by sieving through a 2 mm mesh, DNA was extracted from individual samples using the DNeasy PowerSoil DNA Isolation Kit (QIAGEN, Hilden, Germany) according to the manufacturer’s instructions.

The extracted DNA was used to amplify the V4 region of the 16S rRNA gene (~250 bp) following Bystrianský et al. (2019). Amplification was performed using barcoded primers 515F (GTGTGYCAGCMGCCGCGGTAA) and 806R (CCGGACTACNVGGGTWTCTAAT) (Caporaso et al., 2011; Caporaso et al., 2012). PCR amplification was performed using TP HS DNA-free 2 × Master Mix (Top-Bio, Vestec, Czech Republic) under the following conditions: initial denaturation at 95 °C for 4 min, followed by 25 cycles of denaturation at 94 °C for 45 s, annealing at 50 °C for 30 s, and elongation at 72 °C for 60 s, with a final elongation step at 72 °C for 10 min. Amplicons were purified using the Gel/PCR DNA Fragments Extraction Kit (Geneaid Biotech, Taiwan) according to the manufacturer’s instructions. DNA concentration was measured with the Quantifluor ONE dsDNA System (Promega, USA). Samples were then pooled in equimolar concentrations and sent for sequencing using paired-end (2 × 250 bp) reads on the Illumina MiSeq platform (Illumina, USA) at SeqMe (Czech Republic).^1^ Bioinformatic analyses were conducted using the SEED 2.1.2 pipeline (Větrovský et al., 2018). We assembled the paired-end reads, and removed the low-quality reads, short reads (<250 bp), and long reads (>350 bp). We then clustered the sequences by applying a 97% similarity threshold. Chimeras were identified and excluded using USEARCH. Taxonomic assignment was performed by BLASTing representative OTU sequences against the SILVA 138.1 reference database (edited 2022-03-28), which we used to classify the operational taxonomic units (OTUs) into species and higher taxonomic units. We excluded eukaryotic DNA sequences from the analyses. Further, the OTUs’ abundance in each sample was normalized to the sample with the fewest sequences, particularly 10,537, sequences per sample. Rarefied samples were further standardized to 100%. The sequences assigned to OTUs were aggregated at two taxonomic levels: the species and the genus level. We refer to the first as *species* although bacterial species delineation based on 16S rRNA gene sequences is inherently approximate; the 97% similarity OTU clusters serve as an operational proxy for species, while the taxonomic assignment relies on database matches, which may not always represent true biological species.

To quantify total bacteria, we extracted DNA and evaluated the concentrations (copy numbers) of the rRNA gene in soil samples as a proxy of bacterial biomass quantity (Řezáčová et al., 2021a, 2021b). 16S rRNA gene copy numbers were evaluated in qPCR reactions with Luna Universal Probe qPCR Master Mix (New England Biolabs, Ipswich, MA, USA) according to supplier recommendations. We used the primers Eub338 (ACTCCTACGGGAGGCAGCAG) (Ampe et al., 1999) and Eub518 (ATTACCGCGGCTGCTGG) (Muyzer et al., 1993) and the thermal program as follows: initial denaturation at 95 °C (3 min) and 55 cycles of denaturation at 95 °C (20 s), annealing at 55 °C (20 s), and elongation at 72 °C (25 s). The primers were synthesized and high-performance liquid chromatography-purified at Generi Biotech (Hradec Králové, Czech Republic). The calibration curve was created via measurement of a standard DNA fragment with stock concentration of 3.43 × 10^11^ molecules per microliter, with dilutions ranging from 1:10^9^ to 1:10^4^. The resulting copy number concentrations were expressed per 1 g soil. The analyses were performed on a Roche LightCycler 480 System (Roche Diagnostics, Mannheim, Germany).

Soil pH was measured separately for each pot using a water slurry (1:5, w:v) after 1 h of shaking, with two technical replicates per pot, using a pH meter (Hanna Instruments, Woonsocket, RI, USA).

### Data analyses

Alpha diversity was assessed using taxa richness, Simpson’s diversity index, and Pielou’s evenness (further only evenness), all calculated in PAST v4.11 (Hammer et al., 2001).

To assess how the applied pesticides influenced bacterial and specifically rhizobial diversity, and their overall abundance in soil, we performed a two-way analyses of variance (ANOVAs) with the factors “pesticide” and “soil.” These analyses were conducted on the log-transformed number of genera and on the number of species, Simpson’s diversity index, log-transformed evenness, and log-transformed bacterial abundance, after checking normality using the Shapiro–Wilk test and homogeneity of variance using Levene’s test. *Post-hoc* comparisons were carried out using Tukey’s HSD test to adjust for multiple comparisons. Due to significant interactions between the two factors, we present the results separately for each soil type to facilitate interpretation. To achieve this, we conducted separate one-way ANOVAs with “pesticide” as the factor for each soil type. All statistical analyses were performed using R 4.4.2 (R Core Team, 2022).^2^

Additionally, we evaluated the impact of pesticide application on the composition of bacterial and specifically rhizobial communities, both genus and species level. We first assessed pesticide effects on overall communities across all soils, then analyzed soil-specific impacts. The effects of experimental variables on bacterial communities in pesticide-treated soils were analyzed using redundancy analysis (RDA) in the CANOCO *5.10 for Windows* (Ter Braak and Šmilauer, 2018) and permutational multivariate analysis of variance (PERMANOVA) on Bray–Curtis dissimilarities using adonis2 from the “vegan” R package. Genus and species abundances were square-root transformed prior to analyses.

To evaluate whether the impact of pesticides on bacteria could be influenced by their effect on soil pH, one of the strongest factors affecting microbial communities (Tripathi et al., 2018), we performed a two-way ANOVA on soil pH. Since both factors, “pesticide” and “soil,” significantly interacted, we then conducted one-way analyses of the effect of “pesticide” on soil pH and presented the results separately for each soil type. Additionally, we calculated Pearson correlation coefficients in Microsoft Excel (Microsoft Corporation, USA). Correlations were performed to explore the relationship between pH and total bacterial abundance, pH and the number of genera, pH and evenness and pH and the Simpson diversity index. The effect of pH on community composition was tested using canonical correspondence analysis (CCA) in CANOCO 5.10.

## Results

### Bacterial communities in different soils

**Figure 1:**
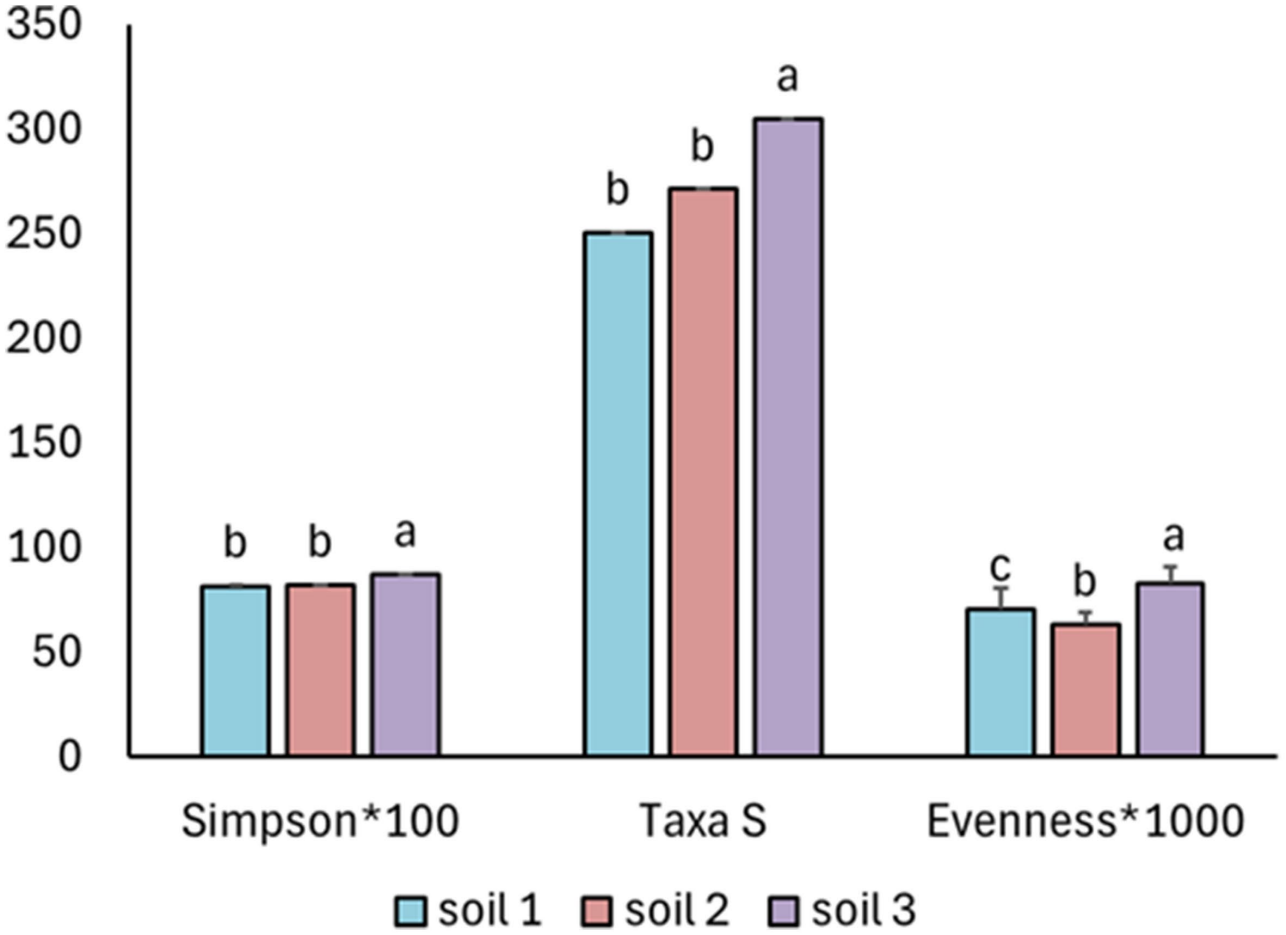
The number of bacterial genera (TaxaS), Simpson’s diversity index, and community evenness (Evenness) for bacteria present in each soil. The bars represent means with standard errors for each soil separately. Different letters above the bars indicate significant differences between means based on one-way ANOVA followed by Tukey’s HSD *post hoc* test (*p* < 0.05); groups sharing the same letter are not significantly different (*n* = 36).

The diversity, taxa richness (genus level), Simpson index, and evenness differed between soils (*F*_2.11_ = 99.6 and *p* = 2.0 × 10^−16^, *F*_2.11_ = 37.4 and *p* = 5.6 × 10^−13^, *F*_2.11_ = 51.1 and *p* = 3.3 × 10^−16^ for taxa richness, Simpson index, and evenness, respectively). Soil 3 consistently exhibited the highest taxa richness, Simpson diversity, and evenness. In contrast, soils 1 and 2 showed lower values for all indices, with no significant differences between them in taxa richness and Simpson index. For evenness, however, soil 1 had significantly higher values than soil 2 (Figure 1).

### The effect of pesticides on bacterial diversity

**Figure 2:**
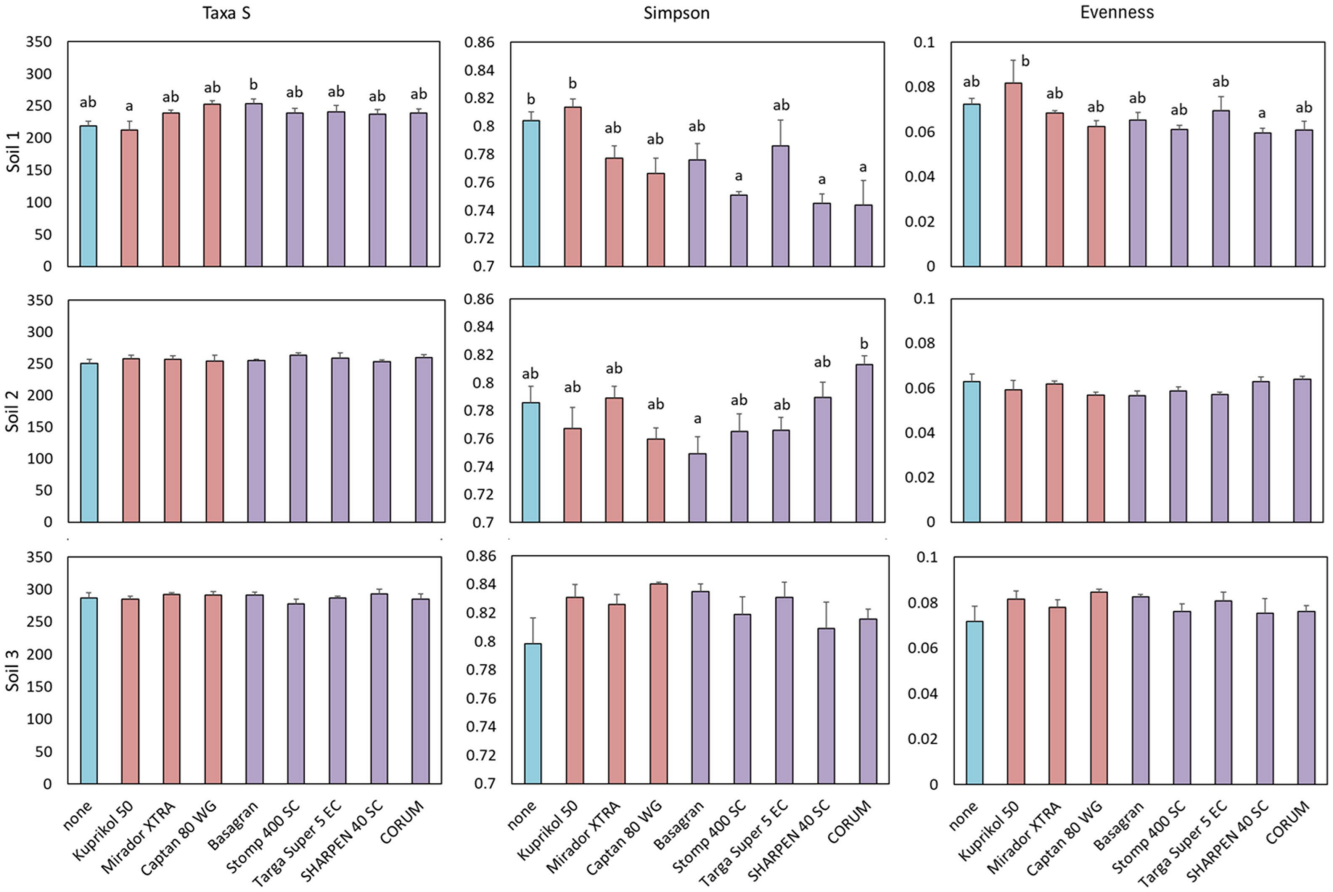
The number of bacterial genera (TaxaS), Simpson’s diversity index, and community evenness (Evenness) for bacteria present in individual soils under the influence of different pesticides. The bars represent means with standard errors for each soil separately. Different letters above the bars indicate significant differences between means based on one-way ANOVA followed by Tukey’s HSD *post hoc* test (*p* < 0.05); groups sharing the same letter are not significantly different (*n* = 4). Where no letters are shown above the bars, the effect of the factor was not significant and the groups do not differ significantly. Colors indicate treatment type: untreated control is highlighted in blue, fungicides are highlighted in pink and herbicides in purple.

The effect of pesticides on bacterial taxa count was significant (*p* < 0.05) only in one of the three soils, soil 1 (Table 3), as indicated by a significant interaction between the factors “pesticide” and “soil” at the species level (*F*_16.81_ = 2.8, *p* = 2 × 10^−16^) and *post hoc* comparisons using Tukey HSD, which confirmed that only in soil 1 specific pesticides significantly differed from the control. In soil 1, two of the tested pesticides, the fungicide Captan 80 WG and the herbicide Basagran, significantly increased bacterial species counts compared to the untreated control (Table S1; Figure S1). Other pesticides generally showed a similar upward trend, although these increases were not statistically significant, and no increase was observed for Kuprikol 50. A similar, though non-significant, pattern was observed at the genus level (Figure 2).

**Table 3:** Significance of the effect of pesticides as revealed by one-way ANOVA on taxa richness at genus level (TaxaS), Simpson diversity indices (Simpson), and community evenness (Evenness) for three analyzed soils.

Used soil	Value type	TaxaS	Simpson	Evenness
Soil 1	F_8.27_	2.7	5.1	2.7
	P	**2.5 × 10** ^**−2**^	**6.6 × 10** ^**−4**^	**2.5 × 10** ^**−2**^
Soil 2	F_8.27_	0.4	3.3	1.6
	P	0.90	**9.4 × 10** ^**−3**^	0.18
Soil 3	F_8.27_	0.7	1.5	1.1
	P	0.70	0.22	0.40

Significant results are highlighted in bold.

Regarding the Simpson’s diversity index, significant differences between pesticides were observed in soils 1 and 2 at both the species and genus levels, which were calculated independently (Table 3, Table S1 and Figure 2, Figure S1). In soil 1, the Simpson index was significantly reduced by the pesticides Stomp 400 SC, Sharpen 40 SC, and CORUM (Figure 2, Figure S1), while other pesticides showed similar but non-significant trends, again with no effect for Kuprikol 50. In soil 2, no significant differences in diversity, as expressed by the Simpson index, were observed between the control and individual pesticides (Figure 2, Figure S1). However, a trend was apparent at the species level, with Basagran and Captan 80 WG showing lower Simpson index values and CORUM showing higher values (Figure S1). A similar trend was also observed at the genus level, although Captan 80 WG did not show a corresponding decrease in diversity at this taxonomic level (Figure 2).Regarding bacterial evenness, no significant effects of pesticides were detected in soils 2 and 3 (Table 3, Table S1; Figure 2, Figure S1). In soil 1, trends relative to the untreated control were observed: Kuprikol 50 tended to increase evenness, while Sharpen 40 SC tended to decrease it at both the species and genus levels. Additionally, Stomp 400 SC showed a decreasing trend at the species level (Figure 2, Figure S1).

### The effect of pesticides on the composition of bacterial communities

**Figure 3:**
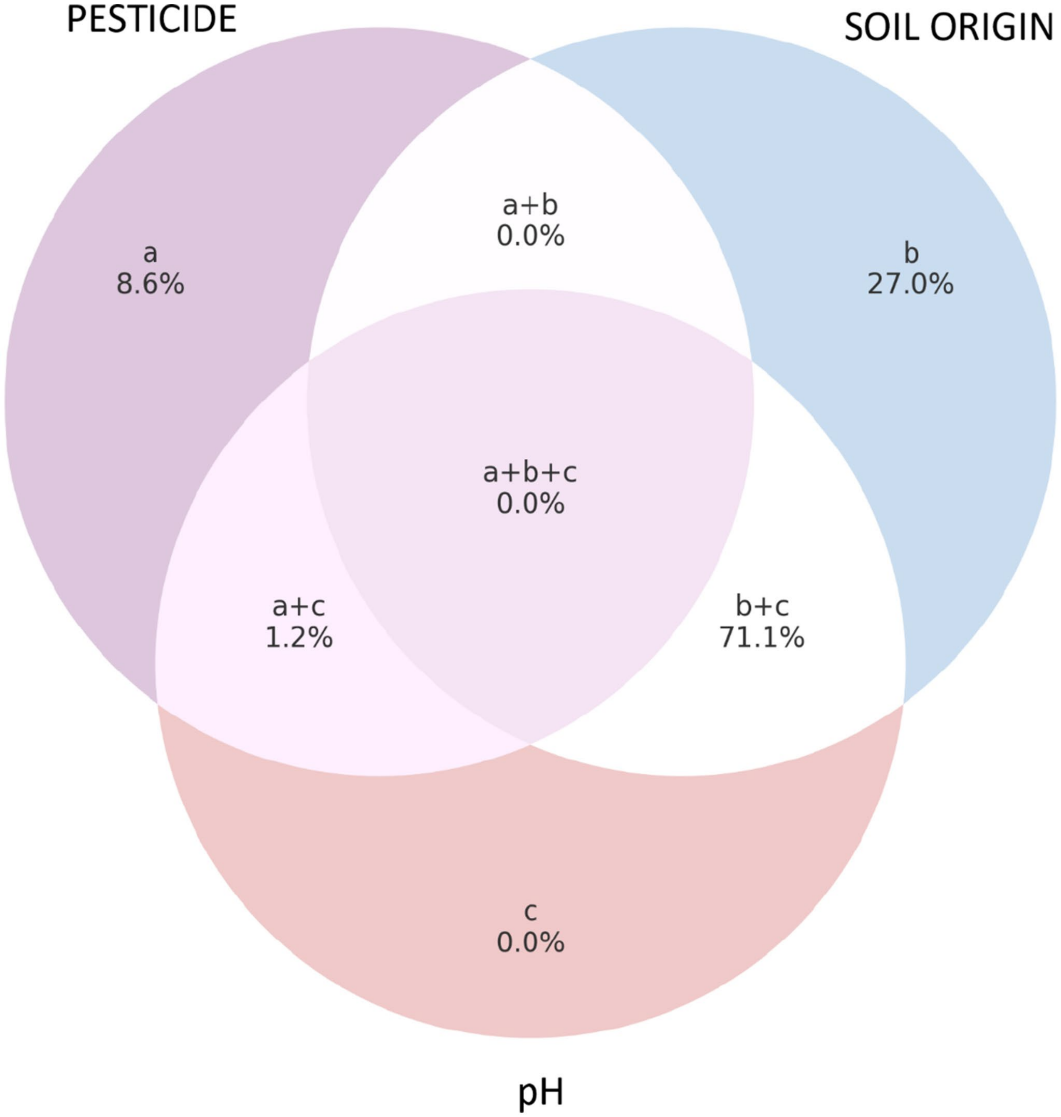
Venn diagram illustrating the partitioning of the total explained variation (%) in soil bacterial communities (genus level) among three groups of predictors [pesticides **(a)**, soil type **(b)**, and soil pH **(c)**] based on redundancy analysis (RDA). The negative values are an artifact of the method and are presented as zero.

Significant differences in the composition of bacterial communities (both the genus and the species level) were observed between the soils used. As revealed by forward selection of environmental variables in RDA and conferred by PERMANOVA, the effect of soil was stronger and explained higher percentage in data variability (Figure 3) than the effect of pesticides, with the effect of pesticides being nonsignificant across all three soils (*p* > 0.05).

When testing each soil individually, the overall effect of pesticides on bacterial communities was significant in all three soils according to PERMANOVA (*p* < 0.05; Table 4). When analyzed by RDA, pesticide effects were significant in soils 1 and 2 (genus level: pseudo-*F* = 4.2, *p* = 0.002 for soil 1; pseudo-*F* = 1.3, *p* = 0.008 for soil 2; species level: pseudo-*F* = 3.7, *p* = 0.002 for soil 1; pseudo-*F* = 1.1, *p* = 0.004 for soil 2), while soil 3 showed a marginally significant effect at the species level (pseudo-*F* = 1.0, *p* = 0.07).

**Table 4:** Effect of pesticides on community compositions of bacteria in soils 1, 2, and 3 at genus and species level.

Used soil	Taxonomic level	Permanova-Bray-Curtis		
		*F*_8.27_	*R* ^2^	*P*
Soil 1	Genus level	3.0	0.469	**0.001**
	Species level	2.6	0.434	**0.001**
Soil 2	Genus level	1.7	0.334	**0.013**
	Species level	1.2	0.259	**0.009**
Soil 3	Genus level	1.6	0.321	**0.014**
	Species level	1.1	0.250	0.06

The table shows the results of permutational multivariate analyses of variance (PERMANOVA). Bolded values indicate statistically significant variables (*p* < 0.05).

Using forward selection of environmental variables, it appeared that in each soil, only some pesticides had a significant effect on the composition of bacterial communities, and the pesticides showing this effect differed between soils. In soil 1, the composition of communities was significantly affected by Kuprikol 50 (genus level: pseudo-*F* = 6.7, *p* = 0.002, species level: pseudo-*F* = 11.0, *p* = 0.002), in soil 2 by CORUM (genus level: pseudo-*F* = 3.0, *p* = 0.002, species level: pseudo-*F* = 1.4, *p* = 0.002) and Captan 80 WG (only genus level: genus level: pseudo-*F* = 2.5, *p* = 0.034), and in soil 3 by Targa Super 5 EC and Stomp 400 SC (only species level: pseudo-*F* = 1.2, *p* = 0.04 and pseudo-*F* = 1.2, *p* = 0.04, respectively).

**Figure 4:**
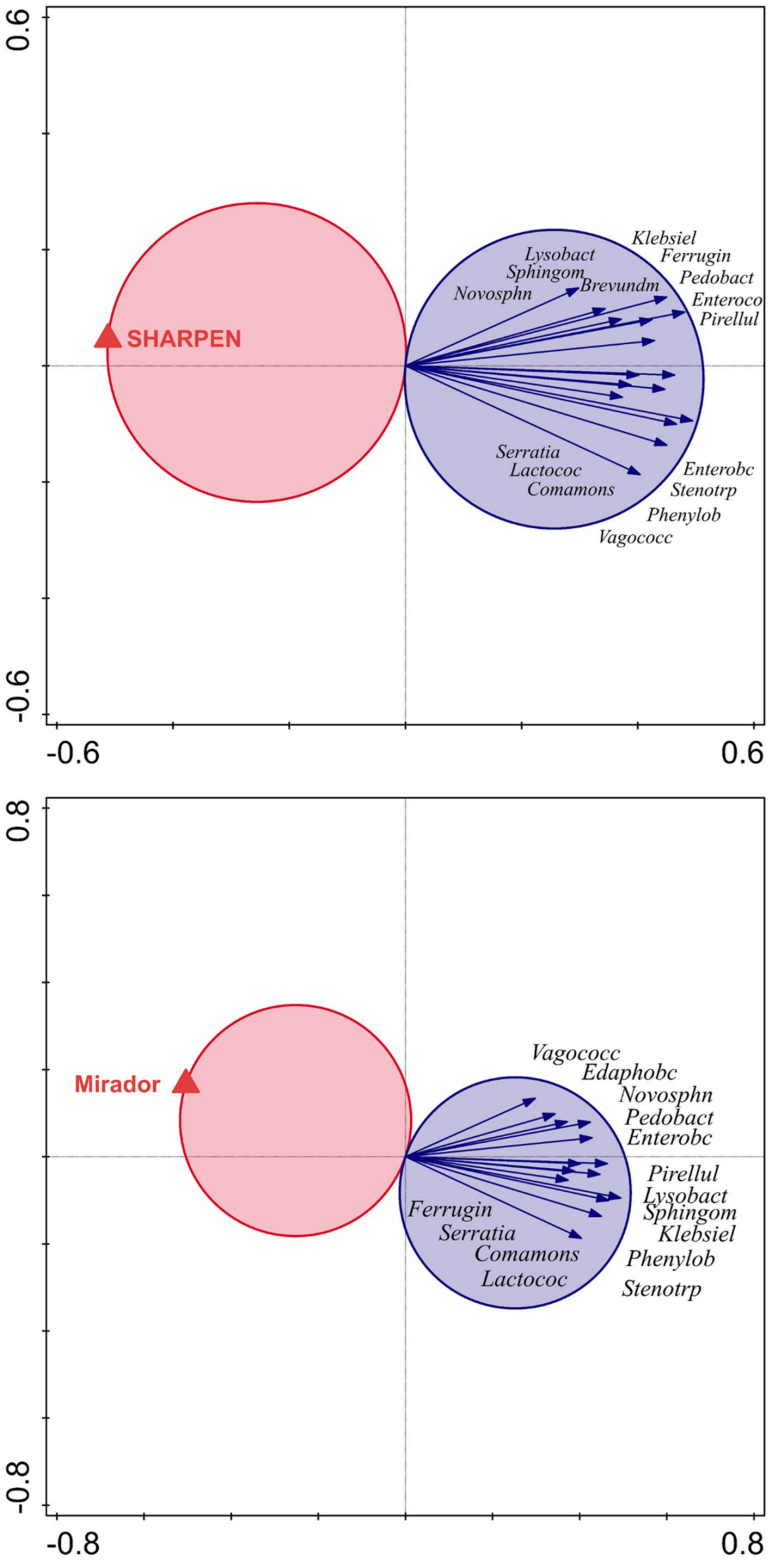
*T*-value biplots from redundancy analysis (RDA) in Canoco showing bacterial genera significantly negatively associated with the pesticides Sharpen 40 SC and Mirador XTRA. These pesticides exhibited either negative or neutral relationships with soil bacterial taxa; only negative associations are shown here. Arrows represent taxa significantly associated (*p* < 0.05) with a given pesticide, as indicated by their position within Van Dobben’s circle. Negative associations are marked by blue circles. The longer arrows indicates stronger and more significant associations. For clarity, taxa with neutral (non-significant) responses were omitted. Genus names are abbreviated; full genus names are listed in Table S2.

**Figure 5:**
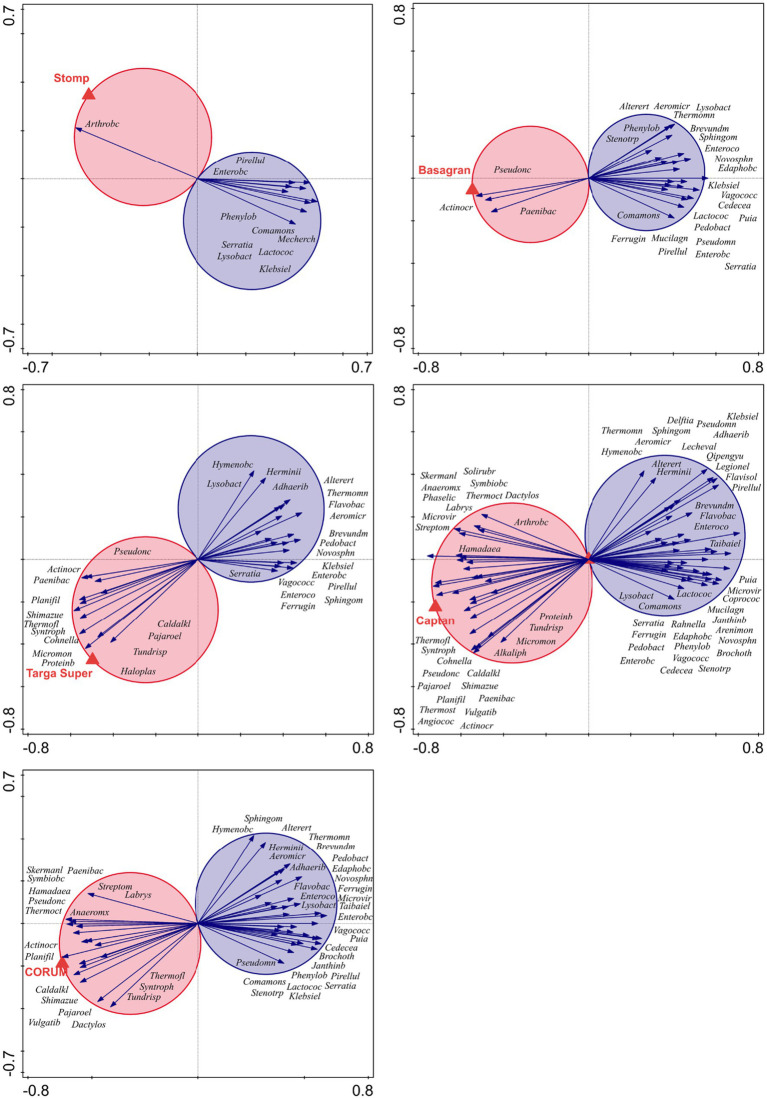
*T*-value biplots from redundancy analysis (RDA) in Canoco displaying bacterial genera significantly associated with pesticides that showed both positive and negative relationships across taxa (Stomp 400 SC, Basagran, Targa Super 5 EC, Captan 80 WG, and CORUM). Arrows represent taxa significantly associated (*p* < 0.05) with a given pesticide, as indicated by their position within van Dobben’s circle. These associations are either positive (red circles) or negative (blue circles). The longer arrows indicates stronger and more significant associations. For clarity, neutral (non-significant) associations are not shown. Genus names are abbreviated; full genus names are listed in Table S2.

For all pesticides except Kuprikol 50, significant associations between the pesticide and specific bacterial genera were found across soils. These associations were either only negative (Sharpen 40 SC and Mirador XTRA, Figure 4) or both negative and positive (Captan 80 WG, Basagran, Stomp 400 SC, CORUM, Targa Super 5 EC, Figure 5). In contrast, when focusing on individual bacterial genera, we found that each genus showed a consistent direction of association across the evaluated pesticides, i.e., no genus was positively associated with some pesticides and negatively with others (Figures 4, 5). Among pathogenic genera, *Klebsiella* and *Serratia* showed the most frequent associations—both were negatively associated with all pesticides (except Kuprikol 50). Among other bacterial ecological groups, genera *Lactococcus*, *Phenylobacterium*, *Vagococcus*, *Pirellula*, *Sphingomonas*, *Novosphingobium*, *Ferruginibacter*, and *Phenylobacterium* showed negative associations with at least five pesticides. In contrast, beneficial bacteria *Paenibacillus* and *Pseudonocardia* displayed positive associations with four pesticides, and several other beneficial bacterial genera also showed positive associations with pesticides (Figures 4, 5).

In addition to assessing the impact of pesticides on the entire bacterial community, we specifically evaluated their effect on rhizobia, crucial nitrogen fixers that play a key role in soil fertility and plant productivity. Across all soil samples, we identified 13 genera and 16 species of rhizobia. The effect of pesticides on their diversity (both, genus and species level)—measured as taxa richness, Simpson’s diversity index and evenness—was not significant (*p* > 0.05). Similarly, pesticides did not have a significant impact (*p* > 0.05) on the community composition of rhizobia as revealed by RDA and PERMANOVA.

### Total bacterial abundance in the soils used in the experiment

**Figure 6:**
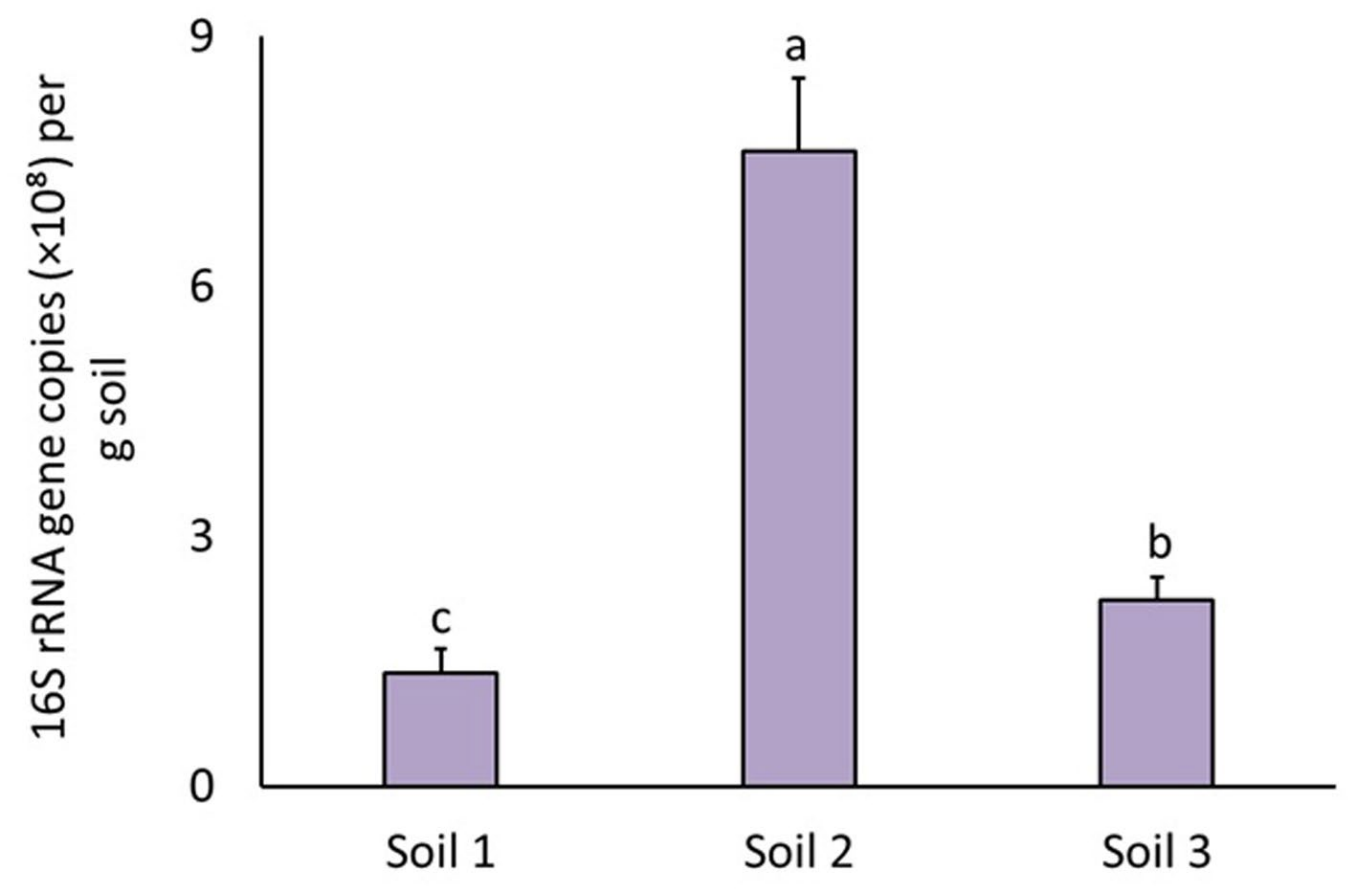
Total bacterial abundance in three different soils expressed as 16S rRNA gene copy numbers per gram of soil. The bars represent means with standard errors. Different letters above the bars indicate significant differences between means based on one-way ANOVA followed by Tukey’s HSD *post hoc* test (*p* < 0.05); groups sharing the same letter are not significantly different (*n* = 36).

Bacterial abundance differed between soils (*F*_2.81_ = 40.0, *p* = 4.91 × 10^−13^). The highest bacterial abundance was found in soil 2, and the lowest in soil 1 (Figure 6).

### The effect of pesticides on total bacterial abundance

**Figure 7:**
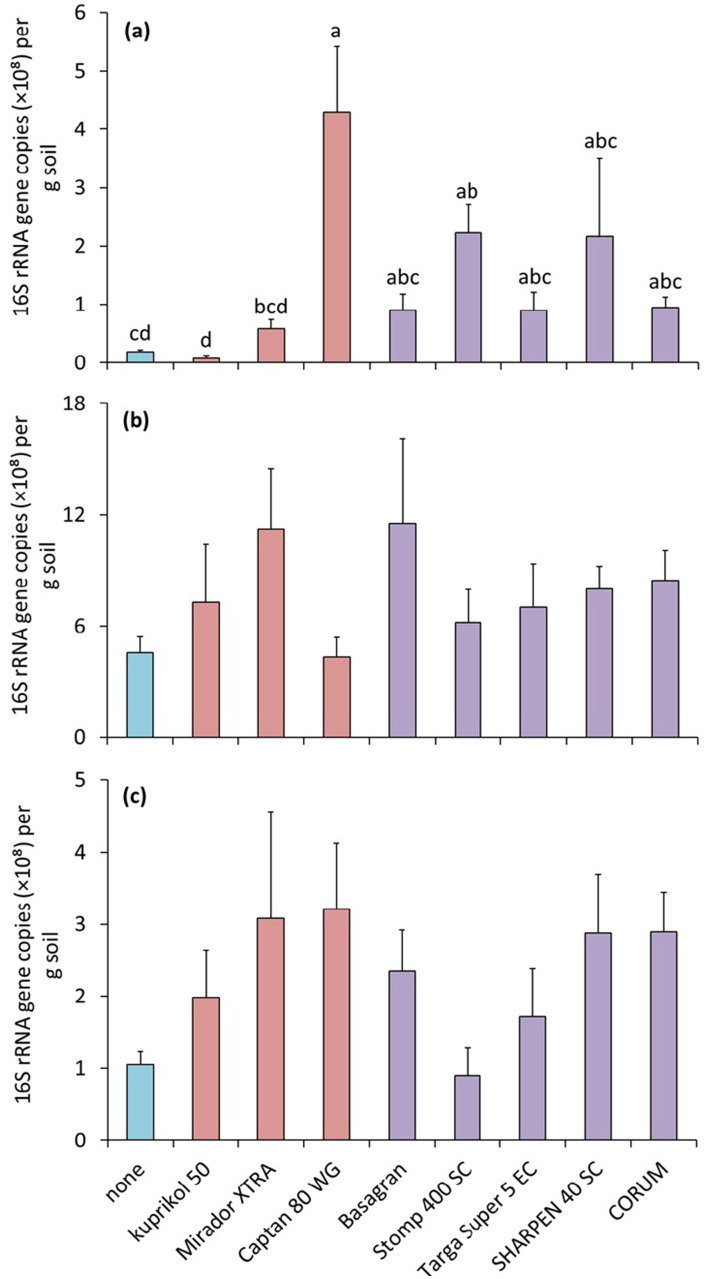
Total bacterial abundance in three different soils (soil 1, **a**; soil 2, **b**; soil 3, **c**) expressed as 16S rRNA gene copy numbers per gram of soil (CN × g^−1^) as affected by pesticides. The bars represent means with standard errors. Different letters above the bars indicate significant difference between means based on one-way ANOVA followed by Tukey’s HSD post hoc test (*p* < 0.05); groups sharing the same letter are not significantly different (*n* = 4). Where no letters are shown above the bars, the effect of the factor was not significant, and the groups do not differ significantly. Untreated control is highlighted in blue, fungicides are highlighted in pink and herbicides in purple.

The effect of pesticides on total bacterial abundance, assessed by the qPCR method, was significant only in soil 1 (*F*_8.27_ = 7.4, *p* = 3.39 × 10^−5^) (Figures 7a–c), which was reflected by a significant interaction between the soil and pesticide factors (*F*_16.81_ = 2.3, *p* = 8.09 × 10^−3^). In this case, the significant effects were observed only for the fungicide Captan 80 WG and the herbicide Stomp 400 SC on bacterial abundance. Both Captan 80 WG and Stomp 400 SC increased bacterial abundance (Figure 7a).

### pH of the soils used

**Figure 8:**
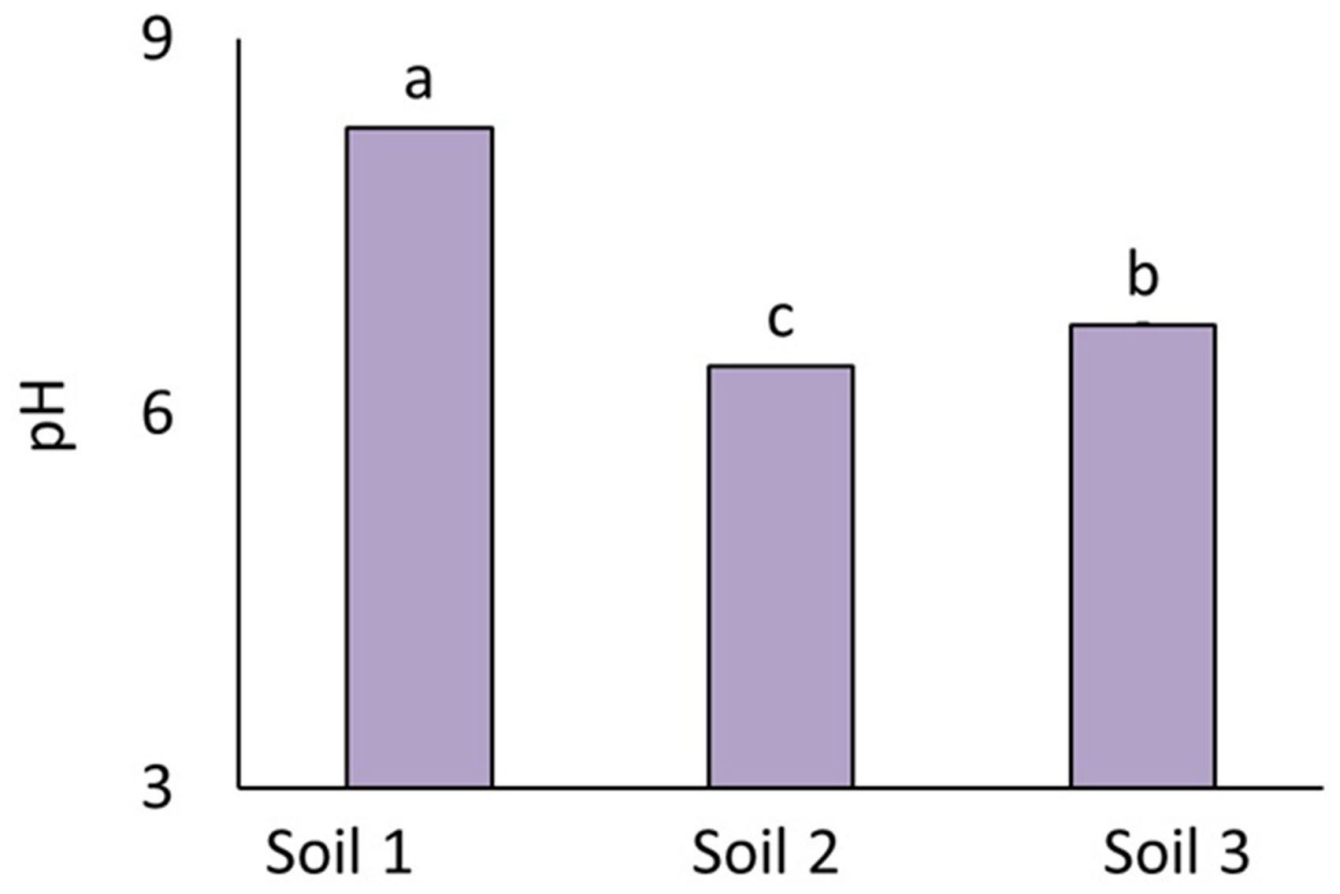
Average pH of the three soils used. The bars represent means with standard errors. Different letters above the bars indicate significant differences between means based on one-way ANOVA followed by Tukey’s HSD *post hoc* test (*p* < 0.05); groups sharing the same letter are not significantly different (*n* = 36).

The pH differed among the three soils used (*F*_2.81_ = 17355.7, *p* = <2 × 10^−16^). The highest pH was recorded in soil 1, and the lowest in soil 2 (Figure 8).

### The effect of pesticides on soil pH

**Figure 9:**
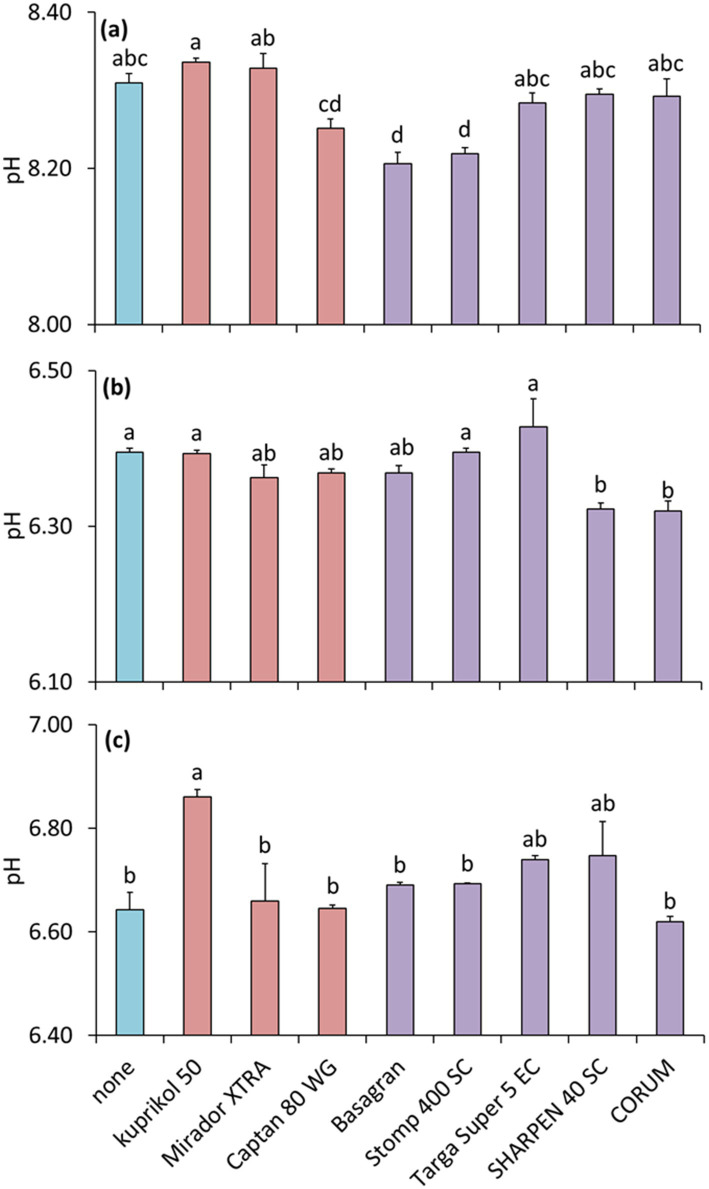
Soil pH of three different soils [soil 1, **(a)**; soil 2, **(b)**; soil 3, **(c)**] as affected by pesticides. The bars represent means with standard errors. Different letters above the bars indicate significant differences between means based on one-way ANOVA followed by Tukey’s HSD *post hoc* test (*p* < 0.05); groups sharing the same letter are not significantly different (*n* = 4). Colors indicate treatment type: Herbicides are highlighted in purple, fungicides in pink, and the untreated control in blue.

The effect of pesticides on pH was dependent on the soil to which the pesticides were applied (the interaction between pesticides and soil was significant, *F*_16.81_ = 4.3, *p* = 5.75 × 10^−6^). In soil 1, the herbicides Basagran and Stomp 400 SC decreased pH compared to the untreated control (*F*_8.27_ = 11.9, *p* = 4.42 × 10^−7^; Figure 9a). In soil 2, the herbicides Sharpen 40 SC and CORUM decreased pH (*F*_8.27_ = 5.8, *p* = 2.42 × 10^−4^; Figure 9b). In soil 3, Kuprikol 50 increased pH (*F*_8.27_ = 4.2, *p* = 2.24 × 10^−3^; Figure 9c).

### The relationship between pH and bacteria

Across all three soils, pH showed a significant (*p* < 0.05) negative correlation with total bacterial abundance (*r* = −0.53689), taxon richness (*r* = −0.5801), and Simpson’s diversity index (*r* = −0.2462) (both latest assessed on genus level).

**Figure 10:**
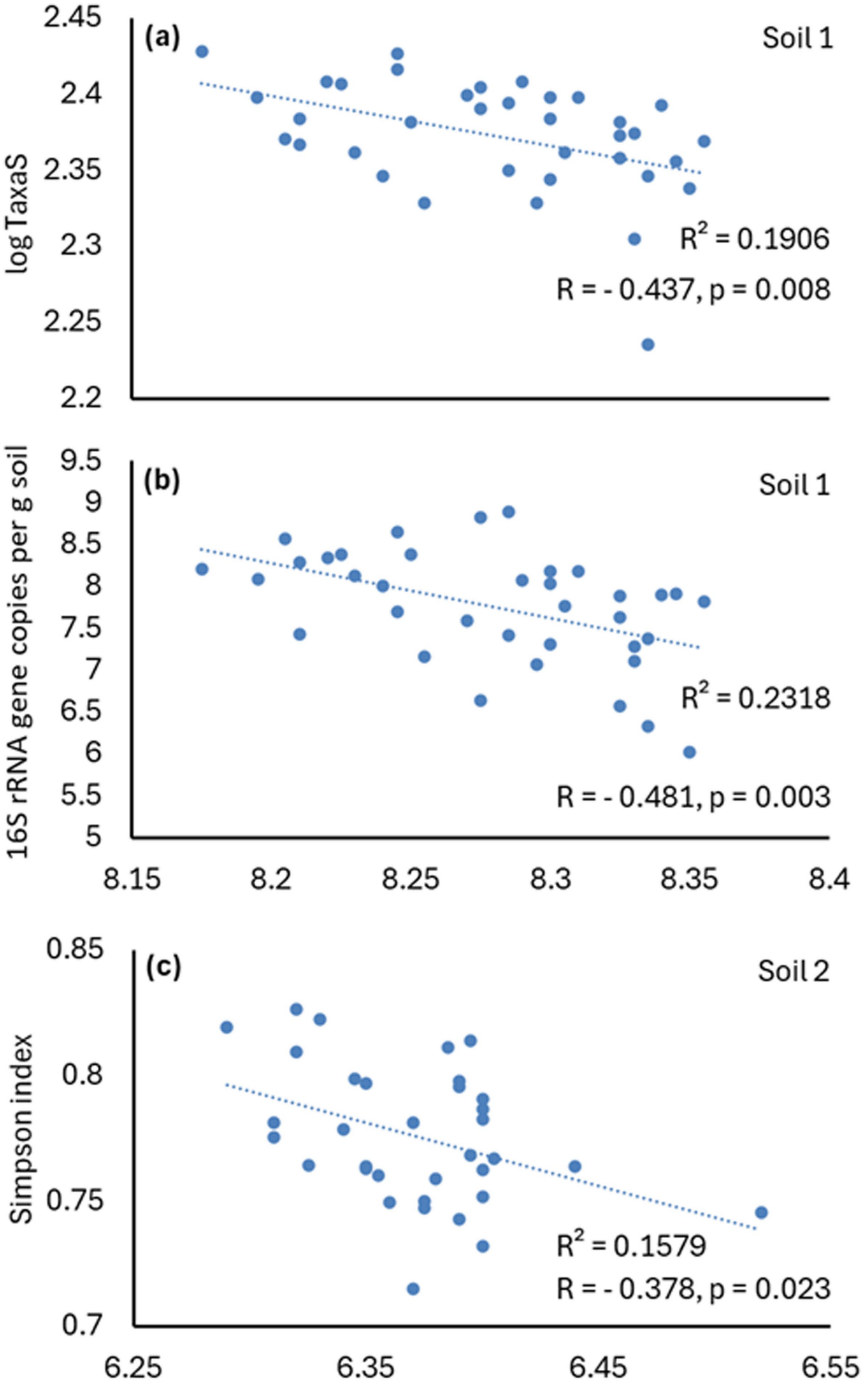
Pearson correlation of taxa richness at the genus level (Taxa S) **(a)** and total bacterial abundance (16S rRNA gene copies per g soil, CN) **(b)** in soil 1, and Simpson index **(c)** in soil 2, with soil pH. Each dot represents a single pot (36 in total: 8 treatments with 4 replicates each, plus 4 control pots). Lines indicate significant Pearson correlations; values of *R*, *R*^2^, and *p*-values are shown for each correlation (*p* < 0.05).

When analyzed each soil separately, no significant correlation (*p* < 0.05) was found in soil 3. The strongest correlation between pH and diversity—measured by taxa richness—was observed in soil 1 (Figure 10a).

In contrast, Simpson’s diversity index and evenness did not correlate with pH in Soil 1. This soil was also the only one where pH significantly correlated with total bacterial abundance (Figure 10b). In Soil 2, pH correlated with Simpson’s diversity index (Figure 10c), but showed no correlation with taxa richness and evenness.

Soil pH also significantly influenced bacterial community composition according to the forward selection of environmental variables (genus level: pseudo-*F* = 57.9, *p* = 0.002; species level: pseudo-*F* = 53.2, *p* = 0.002), and its effect was stronger than that of soil type. According to variation partitioning, the unique contribution of soil pH to explaining variability was small due to its strong correlation with soil type (Figure 3). Nevertheless, pH showed a strong signal and, both on its own and beyond the effect of other variables, had the strongest influence on bacterial communities. The forward selection further indicated that pH explained 60.3% of the total variability in the dataset, whereas soil 3—the only dummy variable from soil type that significantly influenced bacterial community variability—accounted for 23.5%.

## Discussion

This study focused on the impact of five herbicides and three fungicides on bacterial communities in three distinct soils. The aim was to assess how pesticides influence bacterial diversity, community composition, and abundance. Our results indicate that pesticides influence all these key parameters of bacterial communities. Pesticides as a group did not show a uniform effect, with the magnitude and direction of their impact varying between individual products, and no consistent response patterns were detected even within the subcategories of herbicides and fungicides.

The extent of pesticide effects varied primarily between the different soils, with soil pH emerging as a significant factor modulating these impacts. Importantly, RDA including both soil type and pesticide treatment revealed that soil type had a much stronger effect on bacterial community composition than pesticide application. When considering all soils together, the effect of pesticides was not statistically significant (*p* > 0.05), indicating that variation among soil 1, soil 2, and soil 3 overshadowed any subtle shifts caused by pesticide treatments. This highlights the dominant role of soil characteristics in shaping microbial communities and suggests that pesticide effects may be more readily detected when soils are analyzed individually.

### Impact of pesticides on bacteria

Pesticides generally influenced the composition of bacterial communities, although different pesticides were responsible for these effects in different soils. These changes in community composition were not reflected in diversity when measured using species richness, the Simpson diversity index, or community evenness. Conversely, some non-significant changes in community composition were reflected in these diversity metrics. This finding suggests that diversity indices may not always accurately reflect complex ecosystem structures (Jongman et al., 1987).

The fact that pesticide effects on bacterial communities varied among soils suggest that some pesticides may exert taxa-specific impacts depending on soil characteristics. Their effects on bacterial diversity can be either positive or negative, depending on the pesticide used, the soil type, and the index applied to measure diversity.

Biplot analyses revealed that several bacterial genera showed consistent associations with specific pesticides, indicating differential susceptibility or tolerance among taxa. Genera such as *Lactococcus*, *Phenylobacterium*, *Vagococcus*, *Pirellula*, *Sphingomonas*, *Novosphingobium*, *Ferruginibacter*, and *Phenylobacterium* were negatively associated with most of the tested pesticides, suggesting that these taxa may be sensitive to pesticide exposure and could potentially serve as bioindicators of pesticide impact in general. In contrast, genera such as *Paenibacillus*, *Pseudonocardia*, *Shimazuella* or *Arthrobacter* were positively associated with three and four pesticides, respectively, indicating potential tolerance or even metabolic adaptation to these compounds. These genera might therefore serve as indicators of microbial resilience under pesticide treatment. Overall, these findings highlight that pesticide effects on soil bacterial communities are taxon-specific, and that genus-level responses could provide valuable information for assessing soil microbial health and predicting ecological impacts of pesticide applications.

Further, we found that each genus showed a consistent direction of association across all pesticides, i.e., no genus was positively associated with some pesticides and negatively with others. This pattern indicates that the response of soil bacteria to pesticide application followed a similar direction regardless of the specific compound. Among pathogenic genera, *Klebsiella* and *Serratia*, were negatively associated with most pesticides. This uniform negative trend among pathogenic taxa suggests that these bacteria are particularly sensitive to chemical stress or less competitive in pesticide-affected environments. Such a response may reflect the generally inhibitory nature of the tested compounds toward opportunistic or facultatively pathogenic bacteria, which typically thrive under nutrient-rich but chemically stable conditions. In contrast, beneficial soil bacteria such as *Paenibacillus* and *Pseudonocardia* showed only positive associations, each being linked to at least four pesticides, and several other beneficial bacterial genera also displayed positive associations with pesticides. These genera are known for their metabolic versatility, including the ability to degrade complex organic compounds and participate in nutrient cycling (Vainberg et al., 2006; Grady et al., 2016). Their consistent positive association across multiple pesticides may indicate a potential role in pesticide transformation or tolerance, possibly through enzymatic detoxification or the utilization of pesticide residues as alternative carbon sources.

In Soil 1, the pesticides Stomp 400 SC, Sharpen 40 SC, and CORUM were found to reduce bacterial diversity, whereas Captan 80 WG and Basagran increased species richness. For the herbicides Stomp 400 SC and Sharpen 40 SC, both of which contain pendimethalin as the active ingredient, inhibitory effects on certain soil bacteria were previously observed also by Strandberg and Scott-Fordsmand (2004) and Paniagua-López et al. (2023). However, Saha et al. (1991) found that *Azotobacter vinelandii* tolerated pendimethalin and was even able to utilize it as a carbon source. This could explain the positive effects on bacterial diversity or the abundance of specific bacterial taxa that we observed for certain active substances. This idea is further supported by another evidence showing that soil bacteria can metabolize and utilize pesticides as carbon or energy sources (Cook, 1987; Martínez-Toledo et al., 1998; Alatassi et al., 2025).

The effects of the herbicides Basagran and CORUM, both of which contain bentazon, on soil bacteria and their activity were also studied by Allievi et al. (1996), who reported a neutral impact on bacterial communities. Lü et al. (2013) observed that bacterial diversity after application of quizalofop-p-ethyl, also found in the pesticide Targa Super 5 EC, followed a fluctuating trend—initially increasing, then decreasing, and increasing again—before stabilizing on the ninth day. They also identified bacterial species tolerant to quizalofop-p-ethyl. This supports our observation of its neutral effect on overall bacterial diversity and even a positive effect on the abundance of certain bacterial taxa.

In contrast, the pesticide Mirador XTRA had a negative effect on some bacterial taxa in our study. However, Adetutu et al. (2008) reported no impact on bacterial diversity or the structural patterns of the community. This discrepancy could be due to pesticide adherence to soil particles, differences in exposure duration, or simply the presence of a different bacterial community that lacked species negatively affected by this pesticide in our study. According to Wang et al. (2020), azoxystrobin—the active ingredient in Mirador XTRA—has the potential to inhibit nutrient cycling in soil, thereby reducing bacterial abundance.

We observed no detectable effect of the fungicide Kuprikol 50 on bacterial communities. This contrasts with findings by Zhou et al. (2011), who reported negative effects—or at least shifts in community composition—due to copper residues. The difference may be explained by the duration of exposure, as Zhou et al. (2011) assessed effects after 5, 21, 27, 36, and 43 years, suggesting that copper accumulation in soil may have been responsible for the observed changes. Discrepancies between the available data on the active substances included in our study and published literature may also at least partly be explained by differences in soil environments, as even within the three soils included in our experiment, the results varied.

The most pronounced changes in bacterial diversity were observed in Soil 1. In the other two soils, pesticide effects on bacterial diversity were either less significant or absent, possibly due to different interactions between pesticides and the soils’ chemical and biological properties. Soil 1 differed from the other two in pH; it had the highest pH, whereas the others had neutral pH, which is optimal for most bacterial growth and activity (Madigan et al., 2015).

Conversely, even small changes in pH in Soil 1, which already had a relatively high pH prior to pesticide application, could have induced additional stress on bacteria that were already near the limits of their tolerance, potentially impairing their metabolic functions and making them more sensitive to the pesticide’s effects. This could have also altered nutrient availability, further affecting microbial activity.

### The role of pH in the impact of pesticides on bacteria

Our findings show that pesticides influenced soil pH, which in turn affected the composition, diversity, and abundance of bacterial communities, which is consistent with previous reports highlighting pH as a major driver of microbial community structure (Fierer et al., 2007; Constancias et al., 2015; Wan et al., 2020). Most correlations between pH and bacterial parameters were negative, indicating that higher pH was associated with lower bacterial abundance and diversity, in line with previous findings that extreme pH conditions can be unfavorable for bacterial communities (Fierer et al., 2007).

Pesticide application significantly affected soil pH, which is supported by earlier observations (e.g., Stanley et al., 2013). These pH shifts were accompanied by changes in bacterial parameters, suggesting that soil pH may mediate or amplify the magnitude of pesticide impacts on microbial communities. In addition to these indirect effects, pesticide formulations themselves may influence soil chemistry through salts (Wang et al., 2023) or through changes in microbial metabolism that can feed back to community composition and function (Medo et al., 2021).

The causal connection between pH and the pesticide effects is complex and not always straightforward. This can be illustrated using two pesticides, Stomp 40 SC and Sharpen 40 SC, both containing pendimethalin at similar concentrations. In our study, application of these two pesticides resulted in a decrease in pH in soils 1 and 2, respectively, but caused no significant effect on pH in soil 3. Interestingly, in soil 1, application of Stomp 40 SC was associated with an increase in bacterial abundance, whereas Sharpen 40 SC in soil 2 did not show a similar effect. These patterns suggest that pesticide–pH interactions are strongly modulated by soil-specific characteristics and pre-existing microbial communities. Thus, the response of bacterial communities to pesticides is not determined solely by the active ingredient or its formulation, but also by the interplay between pesticide chemistry, soil properties, and microbial context.

### The influence of soil type on bacterial communities

Our results showed that the composition and diversity of bacterial communities differed between soils, and that the effect of soil was stronger than the effect of pesticides. This confirms that soil type is a key factor influencing microbial communities.

Different soils also hosted distinct rhizobial communities, although this variation did not affect their overall diversity. These differences are likely due to variations in the chemical and physical properties of the soil, which were higher for pH, lower for phosphates, and nitrogen and differed in microelement composition in soil 1 compared to the other two soils, and suggest that microbial communities are highly dependent on specific soil conditions. This is consistent with previous studies showing that soil characteristics are a major factor shaping bacterial diversity and function (Nannipieri et al., 2003; Xia et al., 2020; Nikitin et al., 2022).

The distinct bacterial communities found in different soils also provided an opportunity to study the effects of pesticides across varying microbial backgrounds. This allowed us to evaluate whether the impact of individual pesticides is consistent across environments, or whether it depends on the biotic and abiotic conditions of the soil in which they are applied.

### The effect of pesticides on rhizobia and their diversity

Despite the significant impact of pesticides on bacteria in some soils, no significant changes were observed in the diversity of rhizobia—a group of bacteria essential for nitrogen fixation and soil fertility.

These findings suggest that while pesticides can influence general bacterial communities, their effect on specific microorganisms such as rhizobia may be weaker or conditional on other environmental factors.

### Study limitations

This study focused on the short-term response of the soil microbiome 2 weeks after pesticide application—a phase in which DNA from immediately killed cells is no longer detectable, but any potential microbial recovery or adaptation has not yet occurred. Capturing these early effects is crucial, as they may signal microbiome sensitivity to disturbance, affect ecosystem functioning, and potentially foreshadow longer-term changes.

Since the effects of pesticides are often time-dependent—ranging from immediate shifts in microbial composition (within hours to days) to later adaptation or recovery (over weeks to months) (Chang et al., 2023)—extending the observation period could provide additional insight. The half-lives of the tested compounds range from several days to weeks depending on environmental conditions (Peña et al., 2025), which supports the appropriateness of the chosen timeframe for capturing early biological responses.

At the same time, we acknowledge that longer-term experiments may better capture cumulative or persistent effects, representing an important direction for future research.

## Conclusion

This study presents a comprehensive analysis of the effects of a range of herbicides and fungicides on non-target bacterial diversity in soil. It provides novel insights into the effects of certain active substances for which data have been previously unavailable or limited.

Our findings demonstrate that the impact of pesticides on bacterial communities is complex and depends on interactions between the pesticide, soil pH, and soil type. Soil properties—particularly pH—play an important role in mediating pesticide effects on bacterial communities, both directly and indirectly through changes in soil chemistry.

These results indicate that soil pH can modulate the magnitude and even the direction of pesticide effects, emphasizing the need to account for soil-specific characteristics when assessing pesticide safety.

From an ecological and practical perspective, these findings highlight the importance of considering soil-specific conditions in agricultural management. Pesticide selection and application strategies should account for potential impacts on microbial communities, which are crucial for nutrient cycling, soil fertility, and overall ecosystem functioning.

The fact that pesticide effects are strongly defined by the specific soil in which contact occurs should be considered in practice when selecting appropriate active substances.

For more alkaline soils, pesticides such as Basagran or Captan 80 WG may be more suitable, as they showed a positive effect on bacterial diversity under such conditions. In contrast, pendimethalin-based pesticides such as Stomp 400 SC and CORUM are not recommended for alkaline soils, as they significantly reduced the diversity of bacterial communities in that environment.

Kuprikol 50 appears to be suitable for short-term use across a wider range of soil types, as it had no detectable impact on bacterial communities. On the other hand, Sharpen 40 SC and Mirador XTRA consistently exhibited negative effects on bacterial species across all tested soils and therefore cannot be recommended for use.

Surprisingly, the effects of pesticides on soil microbial communities are not fully determined by the identity of the active substance but can also be modulated by other formulation components and their interactions with soil conditions. This is demonstrated by different responses of soil microbial community to two different pesticides sharing the same active substance.

In this study, we focused on the immediate, direct effects of various pesticides on the composition and diversity of soil bacterial communities, with special attention to the rhizobial subgroup. Our results show that while the general microbial community displays sensitivity to pesticide exposure, rhizobia appear to remain relatively unaffected. This resilience suggests that functional microbial groups may respond differently to pesticide stress, highlighting the need to integrate ecological considerations into pesticide management practices.

Future research should focus on the long-term and cumulative impacts of pesticide use on soil microbial networks, ecosystem functions, and sustainable soil health, linking microbial responses to practical recommendations for agricultural practices.

## Data Availability

The datasets presented in this study can be found in the article/Supplementary material and in online repositories/the Sequence Read Archive (NCBI) under PRJNA1108199, SAMN41228747, SRR28959544.

## Footnotes

1
https://www.seqme.eu/en/

2
http://www.R-project.org
