# Supplementary material for: Soil pH mediates the impact of pesticides on bacterial communities, diversity, and abundance
Source: Front Microbiol. 2026 Jan 16;16:1670425. doi: 10.3389/fmicb.2025.1670425 (PMC12858249; doi:10.3389/fmicb.2025.1670425)
Supplement: Supplementary file 1 [file Data_Sheet_1.pdf]

Supporting Information

Article title:

Soil pH Mediates the Impact of Pesticides on Bacterial Communities, Diversity and Abundance

Ema Némethová[1], Milan Řezáč[1], Milan Gryndler[2], Oushadee A J Abeyawardana[1], Veronika Řezáčová[1,*]

[1]Czech Agrifood Research Center, Drnovská 507, Prague 6, Czech Republic

[2]Faculty of Science, J. E. Purkyně University in Ústí nad Labem, České Mládeže 8, 400 96 Ústí nad Labem, Czech Republic

*Author for correspondence: Veronika Řezáčová; Czech Agrifood Research Center, Drnovská 507, Prague 6, Czech Republic; Tel: +420 771 136 025; Email: rezacova@vurv.cz; ORCID: 0000-0002-1749-0355

Table S1. Significance of the effect of pesticides as revealed by one-way ANOVA on putative species richness (TaxaS), Simpson diversity indices (Simpson) and community evenness (Evenness) for three analyzed soils.

		TaxaS	Simpson	Evenness
soil 1	$F_{8.27}$	4.4	5.1	2.9
	P	**1.7 × 10^{-3}**	**6.6 × 10^{-4}**	**0.02**
soil 2	$F_{8.27}$	0.7	3.4	1.8
	P	0.67	**8.4 × 10^{-3}**	0.12
soil 3	$F_{8.27}$	9.7 × 10^{-1}	1.4	9.7 × 10^{-1}
	P	0.48	0.22	0.48

Significant results are highlighted in bold.

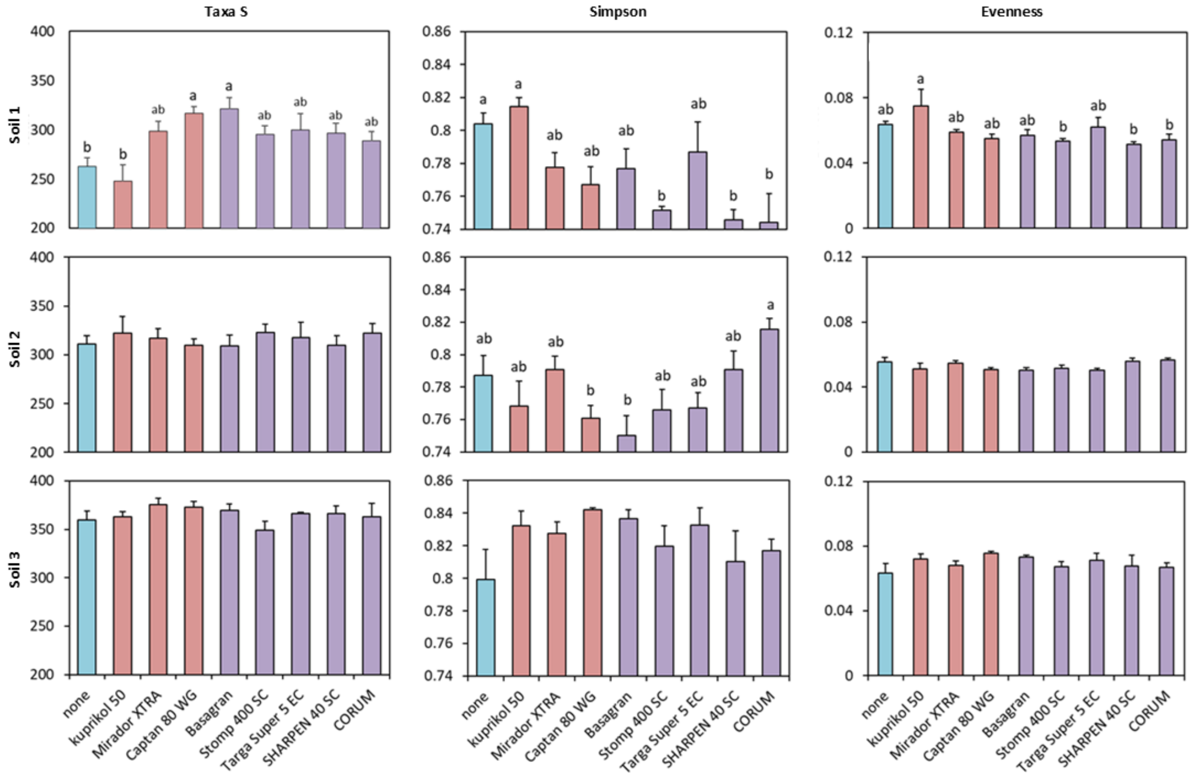

Figure S1. The number of bacterial species (TaxaS), Simpson's diversity index, and community evenness (Evenness) for bacteria present in individual soils under the influence of different pesticides. The bars represent means with standard errors for each soil separately. Different letters above the bars indicate significant differences between means based on on one-way ANOVA followed by Tukey's HSD post hoc test ($p < 0.05$); groups sharing the same letter are not significantly different (n = 4). Where no letters are shown above the bars, the effect of the factor was not significant and the groups do not differ significantly. Untreated control is highlighted in blue, fungicides are highlighted in pink and herbicides in purple.

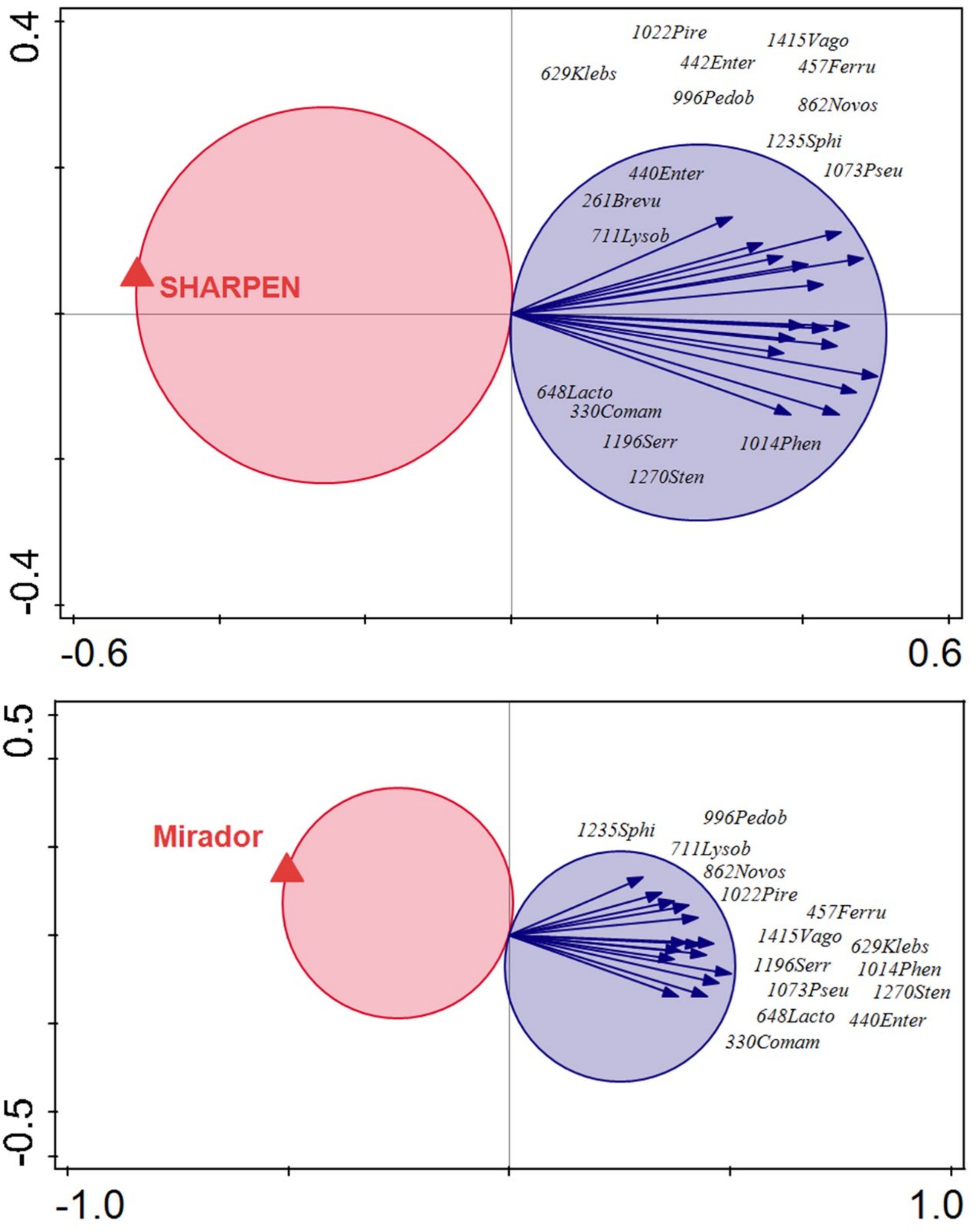

Figure S2. T-value biplots from redundancy analysis (RDA) in Canoco showing bacterial species significantly negatively associated with the pesticides SHARPEN 40 SC and MIRADOR XTRA. These pesticides exhibited either negative or neutral relationships with soil bacterial taxa; only negative associations are shown here. Arrows represent taxa significantly associated ($P < 0.05$) with a given pesticide, as indicated by their position within Van Dobben's circle. Negative associations are marked by blue circles. The longer arrows indicates stronger and more significant associations. For clarity, taxa with neutral (non-significant) responses were omitted. Species names are abbreviated; full species names are listed in Table S3.

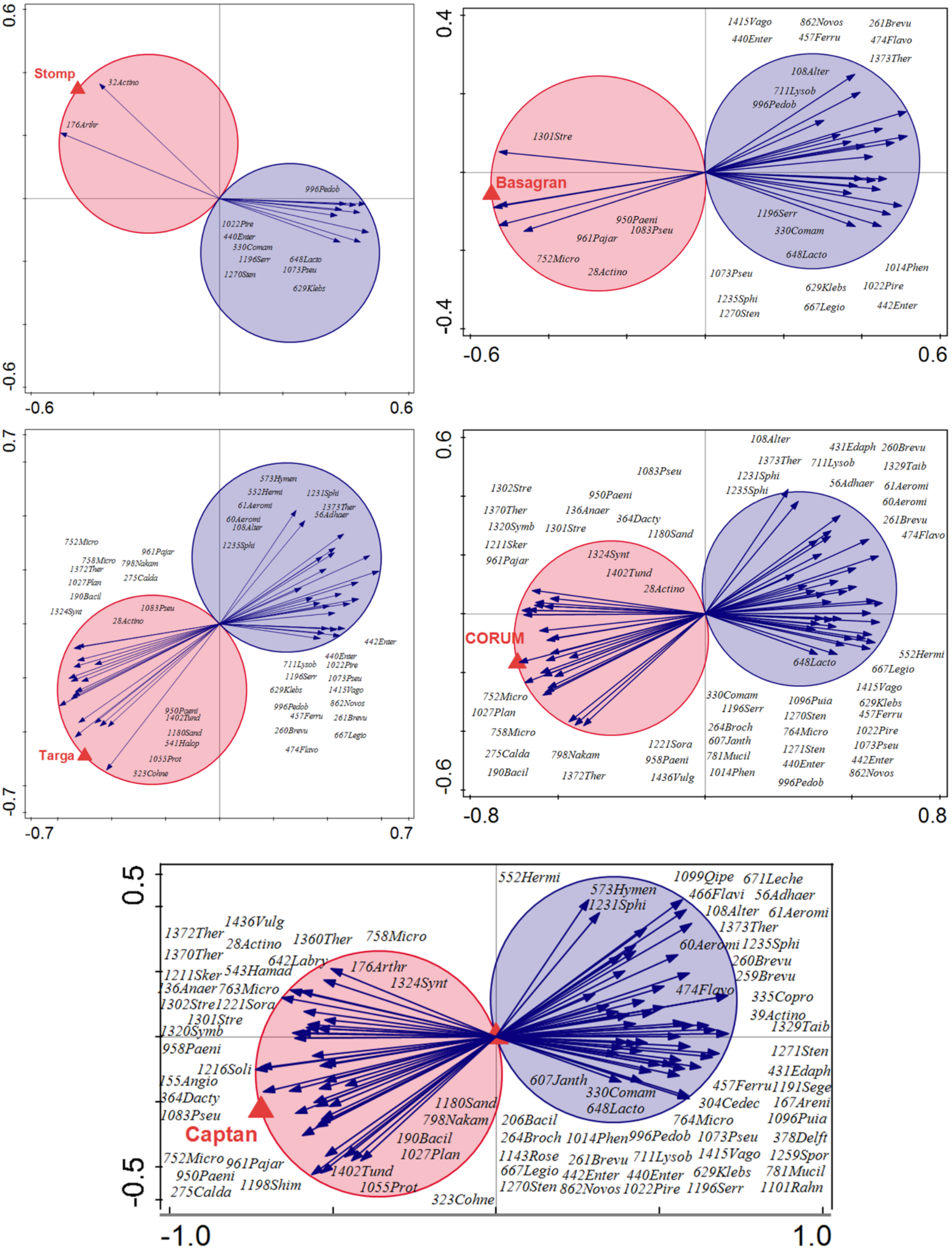
Stomp
Basagran
Targa
CORUM
Captan
0.6
-0.6
0.4
-0.4
0.7
-0.7
0.8
-0.8
0.5
-0.5
-1.0
1.0
552Hermi
573Hymen
1231Sphi
1099Qipe
671Leche
466Flavi
56Adhaer
108Alter
61Aeromi
1373Ther
60Aeromi
1235Sphi
260Brevu
259Brevu
474Flavo
335Copro
39Actino
1329Taib
1271Sten
431Edaph
457Ferru
1191Sege
304Cedec
167Areni
764Micro
1096Puia
1073Pseu
378Delft
1415Vago
1259Spor
629Klebs
781Mucil
1196Serr
1101Rahn
607Janth
330Comam
648Lacto
206Bacil
264Broch
1014Phen
996Pedob
1143Rose
261Brevu
711Lysob
667Legio
442Enter
440Enter
1270Sten
862Novos
1022Pire
1436Vulg
1372Ther
28Actino
1360Ther
758Micro
1370Ther
642Labry
176Arthr
1211Sker
543Hamad
1324Synt
136Anaer
763Micro
1302Stre
1221Sora
1301Stre
1320Symb
958Paeni
1216Soli
155Angio
364Dacty
1083Pseu
1180Sand
798Nakam
190Bacil
1027Plan
752Micro
961Pajar
950Paeni
1402Tund
275Calda
1198Shim
1055Prot
323Cohne

Figure S3. T-value biplots from redundancy analysis (RDA) in Canoco displaying bacterial species significantly associated with pesticides that showed both positive and negative relationships across taxa (Stomp 400 SC, Basagran, Targa Super 5 EC, Captan 80 WG, and CORUM). Arrows represent taxa significantly associated (*P* < 0.05) with a given pesticide, as indicated by their position within Van Dobben's circle. These associations are either positive (red circles) or negative (blue circles). For clarity, neutral (non-significant) associations are not shown. The longer arrows indicates stronger and more significant associations. Species names are abbreviated; full species names are listed in Table S3.

Table S3. The whole species names found three soils which are stated in the T-value biplots in a shortened form

**number**	**species name**
1	Abditibacterium_sp.
2	Acetanaerobacterium_sp.
3	Acetivibrio_sp.
4	Acetobacter_pasteurianus
5	Acetobacterium_bakii
6	Acetomicrobium_sp.
7	Acetonema_sp.
8	Acidibacillus_sp.
9	Acidibacter_sp.
10	Acidicaldus_sp.
11	Acidiphilium_sp.
12	Acidipila-Silvibacterium_sp.
13	Acidisoma_sp.
14	Acidisphaera_sp.
15	Acidothermus_sp.
16	Acidovorax_sp.
17	Acinetobacter_sp.
18	Acinetobacter_ursingii
19	Actinoallomurus_bryophytorum
20	Actinoallomurus_coprocola
21	Actinoallomurus_purpureus
22	Actinoallomurus_radicium
23	Actinoallomurus_sp.
24	Actinoallomurus_yoronensis
25	Actinocatenispora_sera
26	Actinocorallia_herbida
27	Actinocorallia_longicatena
28	Actinocorallia_sp.
29	Actinomadura_adrarensis
30	Actinomadura_livida
31	Actinomadura_napierensis

32	Actinomadura_sp.
33	Actinomadura_sputi
34	Actinomadura_viridilutea
35	Actinomyces_sp.
36	Actinomycetospora_sp.
37	Actinophytocola_gilvus
38	Actinophytocola_sp.
39	Actinophytocola_timorensis
40	Actinoplanes_atraurantiacus
41	Actinoplanes_cibodasensis
42	Actinoplanes_couchii
43	Actinoplanes_derwentensis
44	Actinoplanes_ferrugineus
45	Actinoplanes_globisporus
46	Actinoplanes_nipponensis
47	Actinoplanes_penicillatus
48	Actinoplanes_regularis
49	Actinoplanes_sp.
50	Actinoplanes_utahensis
51	Actinopolymorpha_rutila
52	Actinopolymorpha_sp.
53	Actinorhabdospora_filicis
54	Actinospica_sp.
55	Actinotalea_sp.
56	Adhaeribacter_sp.
57	Adhaeribacter_terrae
58	Aeribacillus_pallidus
59	Aeromicrobium_choanae
60	Aeromicrobium_panaciterrae
61	Aeromicrobium_sp.
62	Aeromonas_bestiarum
63	Aestuariicella_sp.
64	Aetherobacter_rufus
65	Aetherobacter_sp.
66	Afifella_pfennigii
67	Afifella_sp.
68	Afipia_sp.
69	Agaricicola_taiwanensis
70	Agathobacter_sp.
71	Aggregatibacter_sp.
72	Aggregicoccus_edonensis
73	Aggregicoccus_sp.
74	Agromyces_cerinus

75	Agromyces_fucosus
76	Agromyces_humatus
77	Agromyces_iriomotensis
78	Agromyces_sp.
79	Agromyces_ulmi
80	Ahniella_sp.
81	Acholeplasma_hippikon
82	Acholeplasma_sp.
83	Achromobacter_sp.
84	Albimonas_donghaensis
85	Albimonas_pacifica
86	Alcaligenes_sp.
87	Alcanivorax_sp.
88	Algimonas_sp.
89	Alicyclobacillus_acidoterrestris
90	Alicyclobacillus_contaminans
91	Alicyclobacillus_herbarius
92	Alicyclobacillus_macrosporangiidus
93	Alicyclobacillus_pomorum
94	Alicyclobacillus_sendaiensis
95	Alicyclobacillus_sp.
96	Aliivibrio_sp.
97	Alistipes_sp.
98	Alkalibacter_sp.
99	Alkalibaculum_bacchi
100	Alkalicoccus_sp.
101	Alkaliphilus_sp.
102	Alkanindiges_sp.
103	Alloactinosynnema_sp.
104	Allocatelliglobosispora_sp.
105	Allokutzneria_albata
106	Allorhizobium-Neorhizobium-Pararhizobium-Rhizobium_metallidurans
107	Alsobacter_metallidurans
108	Altererythrobacter_sp.
109	Alteribacillus_sp.
110	Alterococcus_sp.
111	Amaricoccus_tamworthensis
112	Aminobacter_sp.
113	Ammoniibacillus_agariperforans
114	Ammoniibacillus_sp.
115	Ammoniphilus_resinae
116	Ammoniphilus_sp.
117	Amnipila_sp.

118	Amphibacillus_xylanus
119	Amycolatopsis_helveola
120	Amycolatopsis_lexingtonensis
121	Amycolatopsis_sacchari
122	Amycolatopsis_sp.
123	Amycolatopsis_xylanica
124	Amylibacter_sp.
125	Anabaena_PCC-7122_sp.
126	Anaerobacillus_sp.
127	Anaerobacterium_chartisolvens
128	Anaerobacterium_sp.
129	Anaerobium_acetethylicum
130	Anaerocolumna_aminovalerica
131	Anaerocolumna_cellulosilytica
132	Anaerocolumna_sp.
133	Anaerocolumna_xylanovorans
134	Anaerofustis_sp.
135	Anaerolinea_sp.
136	Anaeromyxobacter_sp.
137	Anaeroplasma_sp.
138	Anaerosalibacter_bizertensis
139	Anaerosalibacter_sp.
140	Anaerosinus_sp.
141	Anaerosolibacter_sp.
142	Anaerospora_sp.
143	Anaerosporobacter_sp.
144	Anaerosporomusa_sp.
145	Anaerosporomusa_subterranea
146	Anaerostignum_sp.
147	Anaerostipes_sp.
148	Anaerotruncus_sp.
149	Anaerovorax_sp.
150	Anaplasma_marginale
151	Aneurinibacillus_migulanus
152	Aneurinibacillus_soli
153	Aneurinibacillus_sp.
154	Aneurinibacillus_thermoaerophilus
155	Angiococcus_sp.
156	Angustibacter_luteus
157	Angustibacter_sp.
158	Antricoccus_sp.
159	Apibacter_sp.
160	Aquamicrobium_sp.

161	Aquicella_sp.
162	Aquisphaera_giovannonii
163	Aquisphaera_sp.
164	Arboricoccus_pini
165	Arboricoccus_sp.
166	Arcticibacter_sp.
167	Arenimonas_sp.
168	Arenimonas_subflava
169	Archangium_sp.
170	Aridibacter_sp.
171	Arsenicitalea_sp.
172	Arthrobacter_alpinus
173	Arthrobacter_deserti
174	Arthrobacter_ginkgonis
175	Arthrobacter_psychrochitiniphilus
176	Arthrobacter_sp.
177	Asanoa_endophytica
178	Asanoa_sp.
179	Asticcacaulis_sp.
180	Aurantisolimonas_sp.
181	Aureimonas_altamirensis
182	Aureimonas_sp.
183	Azoarcus_sp.
184	Azospira_sp.
185	Azospirillum_brasilense
186	Azospirillum_canadense
187	Bacillus_alcalophilus
188	Bacillus_alkalitelluris
189	Bacillus_anthracis
190	Bacillus_aryabhattai
191	Bacillus_asahii
192	Bacillus_azotoformans
193	Bacillus_badius
194	Bacillus_benzoevorans
195	Bacillus_cereus
196	Bacillus_circulans
197	Bacillus_clausii
198	Bacillus_coagulans
199	Bacillus_decolorationis
200	Bacillus_drentensis
201	Bacillus_firmus
202	Bacillus_flexus
203	Bacillus_ginsengihumi

204	Bacillus_glycinifermentans
205	Bacillus_graminis
206	Bacillus_halodurans
207	Bacillus_horti
208	Bacillus_chandigarhensis
209	Bacillus_infernus
210	Bacillus_lentus
211	Bacillus_licheniformis
212	Bacillus_litoralis
213	Bacillus_malikii
214	Bacillus_megaterium
215	Bacillus_mycoides
216	Bacillus_niacini
217	Bacillus_oceanisediminis
218	Bacillus_oryzaecorticis
219	Bacillus_plakortidis
220	Bacillus_psychrosaccharolyticus
221	Bacillus_pumilus
222	Bacillus_purgationiresistens
223	Bacillus_rubiinfantis
224	Bacillus_ruris
225	Bacillus_salitolerans
226	Bacillus_soli
227	Bacillus_sp.
228	Bacillus_thermolactis
229	Bacillus_thuringiensis
230	Bacillus_tianshenii
231	Bacillus_weihaiensis
232	Bacteriovorax_sp.
233	Bacteroides_sp.
234	Baia_sp.
235	Bauldia_sp.
236	Bdellovibrio_bacteriovorus
237	Bdellovibrio_exovorus
238	Bdellovibrio_sp.
239	Bergeyella_sp.
240	Bifidobacterium_angulatum
241	Bifidobacterium_dentium
242	Bizionia_argentinensis
243	Blastocatella_sp.
244	Blastococcus_sp.
245	Blastopirellula_sp.
246	Blautia_sp.

247	Bosea_eneae
248	Bosea_sp.
249	Bradyrhizobium_sp.
250	Brachybacterium_nesterenkovii
251	Brassicibacter_sp.
252	Brevibacillus_borstelensis
253	Brevibacillus_ginsengisoli
254	Brevibacillus_laterosporus
255	Brevibacillus_limnophilus
256	Brevibacillus_sp.
257	Brevibacillus_thermoruber
258	Brevibacterium_sp.
259	Brevundimonas_diminuta
260	Brevundimonas_lenta
261	Brevundimonas_sp.
262	Breznakia_sp.
263	Brockia_sp.
264	Brochothrix_thermosphacta
265	Bryobacter_sp.
266	Budvicia_aquatica
267	Buchnera_aphidicola
268	Burkholderia-Caballeronia-Paraburkholderia_cenocepacia
269	Burkholderia-Caballeronia-Paraburkholderia_pseudomallei
270	Burkholderia-Caballeronia-Paraburkholderia_sp.
271	Byssovorax_sp.
272	Caedibacter_sp.
273	Caedibacter_varicaedens
274	Caenimonas_sp.
275	Caldalkalibacillus_sp.
276	Caldalkalibacillus_thermarum
277	Caldibacillus_sp.
278	Caldicoprobacter_sp.
279	Caldisericum_sp.
280	Calditerricola_yamamurae
281	Caloramator_mitchellensis
282	Caloramator_sp.
283	Calothrix_NIES-2100_sp.
284	Caminicella_sp.
285	Capnocytophaga_sp.
286	Caproiciproducens_galactitolivorans
287	Caproiciproducens_sp.
288	Caryophanon_sp.
289	Caryophanon_tenue

290	Castellaniella_sp.
291	Catellatospora_bangladeshensis
292	Catellatospora_sp.
293	Catellatospora_tagetis
294	Catelliglobosispora_koreensis
295	Catenisphaera_sp.
296	Catenulispora_acidiphila
297	Catenulispora_yoronensis
298	Catenuloplanes_atrovinosus
299	Caulobacter_fusiformis
300	Caulobacter_sp.
301	Cavicella_sp.
302	Cavicella_subterranea
303	Cedecea_neteri
304	Cedecea_sp.
305	Cellulomonas_composti
306	Cellulomonas_soli
307	Cellulomonas_sp.
308	Cellulosilyticum_lentocellum
309	Cellulosilyticum_sp.
310	Cellulosimicrobium_cellulans
311	Cellvibrio_sp.
312	Cephaloticoccus_sp.
313	Citricoccus_sp.
314	Citrifermentans_sp.
315	Citrobacter_amalonaticus
316	Citrobacter_sp.
317	Cloacibacterium_sp.
318	Clostridioides_mangenotii
319	Clostridium_sensu_stricto_sp.
320	Cnuella_sp.
321	Cohnella_hongkongensis
322	Cohnella_saccharovorans
323	Cohnella_sp.
324	Cohnella_thermotolerans
325	Cohnella_xylanilytica
326	Cohnella_yongneupensis
327	Colidextribacter_sp.
328	Collinsella_sp.
329	Comamonas_sp.
330	Comamonas_testosteroni
331	Commensalibacter_sp.
332	Conexibacter_sp.

333	Conexibacter_woesei
334	Conyzicola_nivalis
335	Coprococcus_sp.
336	Coprothermobacter_sp.
337	Corallococcus_coralloides
338	Corynebacterium_atrinae
339	Corynebacterium_massiliense
340	Corynebacterium_pilbarense
341	Corynebacterium_sp.
342	Coxiella_burnetii
343	Coxiella_sp.
344	Crassaminicella_profunda
345	Craurococcus-Caldovatus_roseus
346	Craurococcus-Caldovatus_sp.
347	Crenobacter_sp.
348	Crenothrix_sp.
349	Crinalium_SAG_22.89_sp.
350	Crocinitomix_sp.
351	Crossiella_sp.
352	Cryobacterium_psychrotolerans
353	Cryptanaerobacter_sp.
354	Cupriavidus_sp.
355	Curtobacterium_herbarum
356	Curvibacter_sp.
357	Cutibacterium_sp.
358	Cycloclasticus_sp.
359	Cylindrospermum_PCC-7417_sp.
360	Cylindrospermum_SAG_11.82_sp.
361	Cystobacter_gracilis
362	Cystobacter_velatus
363	Cytophaga_sp.
364	Dactylosporangium_matsuzakiense
365	Dactylosporangium_sp.
366	Defluviicoccus_sp.
367	Defluviitalea_sp.
368	Dehalobacter_sp.
369	Dehalobacterium_formicoaceticum
370	Dehalobacterium_sp.
371	Dehalococcoides_sp.
372	Dechloromonas_sp.
373	Deinococcus_alpinitundrae
374	Deinococcus_budaensis
375	Deinococcus_ficus

376	Deinococcus_soli
377	Deinococcus_sp.
378	Delftia_acidovorans
379	Demequina_sp.
380	Desertibacter_roseus
381	Desmonostoc_PCC-6302_sp.
382	Desulfallas-Sporotomaculum_arcticus
383	Desulfallas-Sporotomaculum_gibsoniae
384	Desulfallas-Sporotomaculum_sp.
385	Desulfallas-Sporotomaculum_syntrophicum
386	Desulfallas-Sporotomaculum_thermosapovorans
387	Desulfatiglans_sp.
388	Desulfitibacter_sp.
389	Desulfitispora_sp.
390	Desulfitobacterium_dehalogenans
391	Desulfitobacterium_dichloroeliminans
392	Desulfitobacterium_metallireducens
393	Desulfitobacterium_sp.
394	Desulfobacca_sp.
395	Desulfobulbus_sp.
396	Desulfocapsa_sp.
397	Desulfoconvexum_sp.
398	Desulfofarcimen_intricatum
399	Desulfofarcimen_sp.
400	Desulfohalotomaculum_peckii
401	Desulfohalotomaculum_sp.
402	Desulfonispora_thiosulfatigenes
403	Desulfopila_sp.
404	Desulfosporosinus_sp.
405	Desulfosporosinus_youngiae
406	Desulfotomaculum_ferrireducens
407	Desulfotomaculum_ruminis
408	Desulfotomaculum_sp.
409	Desulfovibrio_sp.
410	Desulfovirga_sp.
411	Desulfuribacillus_sp.
412	Desulfurispora_sp.
413	Desulfuromonas_sp.
414	Dethiobacter_sp.
415	Devosia_geojensis
416	Devosia_neptuniae
417	Devosia_sp.
418	Dietzia_sp.

419	Dinghuibacter_sp.
420	Dokdonella_sp.
421	Domibacillus_sp.
422	Dongia_sp.
423	Duganella_sp.
424	Dyadobacter_beijingensis
425	Dyadobacter_koreensis
426	Dyadobacter_psychrophilus
427	Dyadobacter_sp.
428	Dysgonomonas_sp.
429	Edaphobacter_sp.
430	Edaphobaculum_flavum
431	Edaphobaculum_sp.
432	Effusibacillus_sp.
433	Egicoccus_sp.
434	Eisenbergiella_sp.
435	Emticicia_sp.
436	Endomicrobium_sp.
437	Ensifer_adhaerens
438	Ensifer_meliloti
439	Enterobacter_cloacae
440	Enterobacter_sp.
441	Enterococcus_gallinarum
442	Enterococcus_sp.
443	Enterorhabdus_sp.
444	Epulopiscium_sp.
445	Erysipelatoclostridium_sp.
446	Erysipelothrix_sp.
447	Erythrobacter_sp.
448	Escherichia-Shigella_coli
449	Ethanoligenens_harbinense
450	Ethanoligenens_sp.
451	Eubacterium_sp.
452	Euzebya_sp.
453	Exiguobacterium_sp.
454	Falsirhodobacter_sp.
455	Falsochrobactrum_sp.
456	Ferruginibacter_profundus
457	Ferruginibacter_sp.
458	Fibrella_sp.
459	Fictibacillus_phosphorivorans
460	Filomicrobium_sp.
461	Fimbriiglobus_sp.

462	Finegoldia_sp.
463	Flaviaesturariibacter_sp.
464	Flaviflexus_sp.
465	Flavihumibacter_sp.
466	Flavisolibacter_sp.
467	Flavisolibacter_tropicus
468	Flavitalea_gansuensis
469	Flavitalea_sp.
470	Flavobacterium_frigidimaris
471	Flavobacterium_hercynium
472	Flavobacterium_psychrophilum
473	Flavobacterium_saliperosum
474	Flavobacterium_sp.
475	Flavobacterium_verecundum
476	Flavonifractor_sp.
477	Flexibacter_sp.
478	Flexivirga_lutea
479	Flindersiella_endophytica
480	Fluviicola_sp.
481	Fontibacillus_sp.
482	Fonticella_sp.
483	Fontimonas_sp.
484	Francisella_sp.
485	Frankia_inefficax
486	Frankia_sp.
487	Friedmanniella_sp.
488	Frigoribacterium_sp.
489	Frischella_sp.
490	Frisingicoccus_sp.
491	Fulvivirga_sp.
492	Fusibacter_sp.
493	Gaiella_occulta
494	Gaiella_sp.
495	Galbitalea_sp.
496	Gallicola_sp.
497	Gallionella_sp.
498	Garciella_sp.
499	Gelria_sp.
500	Geminicoccus_sp.
501	Gemmata_sp.
502	Gemmatimonas_sp.
503	Gemmatirosa_kalamazoonesis
504	Gemmatirosa_sp.

505	Gemmobacter_sp.
506	Geoalkalibacter_sp.
507	Geobacillus_sp.
508	Geobacillus_subterraneus
509	Geobacillus_thermodenitrificans
510	Geobacter_sp.
511	Geodermatophilus_bullaregiensis
512	Geodermatophilus_daqingensis
513	Geodermatophilus_sp.
514	Georgenia_muralis
515	Georgenia_soli
516	Georgenia_sp.
517	Geosporobacter_ferrireducens
518	Geosporobacter_sp.
519	Geotalea_sp.
520	Geothermomicrobium_sp.
521	Geothermomicrobium_terrae
522	Gilliamella_apicola
523	Globicatella_sp.
524	Glycomyces_sp.
525	Gordonia_lacunae
526	Gottschalkia_acidurici
527	Gottschalkia_sp.
528	Gracilibacillus_halotolerans
529	Gracilibacter_sp.
530	Haemophilus_pittmaniae
531	Haliangium_sp.
532	Haliscomenobacter_sp.
533	Haloactinopolyspora_sp.
534	Halobacillus_sp.
535	Halocella_sp.
536	Haloferula_sp.
537	Halolactibacillus_sp.
538	Halomonas_sp.
539	Halomonas_venusta
540	Halopeptonella_sp.
541	Haloplasma_sp.
542	Halovulum_sp.
543	Hamadaea_sp.
544	Hasllibacter_sp.
545	Hassallia_sp.
546	Hazenella_sp.
547	Helcococcus_sp.

548	Herbaspirillum_sp.
549	Herbinix_hemicellulosilytica
550	Herbinix_luporum
551	Herbinix_sp.
552	Herminiimonas_sp.
553	Hespellia_sp.
554	Hirschia_sp.
555	Holophaga_sp.
556	Holospora_sp.
557	Humibacillus_xanthopallidus
558	Hungateiclostridium_clariflavum
559	Hungateiclostridium_sp.
560	Hungateiclostridium_straminisolvens
561	Hydrocarboniphaga_effusa
562	Hydrogenibacillus_schlegelii
563	Hydrogenispora_ethanolica
564	Hydrogenispora_sp.
565	Hydrogenoanaerobacterium_sp.
566	Hydrogenophaga_sp.
567	Hylemonella_gracilis
568	Hymenobacter_algoricola
569	Hymenobacter_gelipurpurascens
570	Hymenobacter_lapidarius
571	Hymenobacter_psychrophilus
572	Hymenobacter_saemangeumensis
573	Hymenobacter_sp.
574	Hymenobacter_terrae
575	Hyphomicrobium_sp.
576	Chiayiivirga_sp.
577	Chitinophaga_rupis
578	Chitinophaga_soli
579	Chitinophaga_sp.
580	Chloronema_sp.
581	Chondromyces_apiculatus
582	Chondromyces_crocatus
583	Chondromyces_pediculatus
584	Chryseobacterium_indologenes
585	Chryseobacterium_lathyri
586	Chryseobacterium_sp.
587	Chryseobacterium_yeoncheonense
588	Chryseolinea_sp.
589	Chthoniobacter_sp.
590	Chthonomonas_sp.

591	Chungangia_koreensis
592	Iamia_sp.
593	Idiomarina_sp.
594	Ileibacterium_valens
595	Ilumatobacter_fluminis
596	Ilumatobacter_sp.
597	Immundisolibacter_sp.
598	Inquilinus_limosus
599	Inquilinus_sp.
600	Intestinibacter_sp.
601	Intrasporangium_sp.
602	Irregularibacter_muris
603	Irregularibacter_sp.
604	Jahnella_sp.
605	Jahnella_thaxteri
606	Janibacter_melonis
607	Janthinobacterium_sp.
608	Janthinobacterium_svalbardensis
609	Jatrophihabitans_sp.
610	Jeotgalibacillus_marinus
611	Jeotgalicoccus_halophilus
612	Jiangella_alkaliphila
613	Jiangella_sp.
614	Jonesia_denitrificans
615	Kaistia_sp.
616	Keratinibaculum_sp.
617	Ketobacter_sp.
618	Kibdelosporangium_banguiense
619	Kineococcus_endophyticus
620	Kineococcus_sp.
621	Kineococcus_xinjiangensis
622	Kineosphaera_limosa
623	Kineosporia_aurantiaca
624	Kineosporia_rhamnosa
625	Kineosporia_sp.
626	Kitasatospora_gansuensis
627	Kitasatospora_sp.
628	Klebsiella_oxytoca
629	Klebsiella_pneumoniae
630	Klebsiella_sp.
631	Klenkia_sp.
632	Knoellia_sp.
633	Kocuria_sp.

634	Kocuria_varians
635	Kouleothrix_sp.
636	Kribbella_ginsengisoli
637	Kribbella_sp.
638	Kroppenstedtia_eburnea
639	Kroppenstedtia_sp.
640	Ktedonobacter_sp.
641	Labrys_methylaminiphilus
642	Labrys_sp.
643	Laceyella_tengchongensis
644	Lacibacter_cauensis
645	Lacibacter_sp.
646	Lactobacillus_delbrueckii
647	Lactobacillus_sp.
648	Lactococcus_lactis
649	Lactococcus_sp.
650	Lacunisphaera_sp.
651	Lachnoclostridium_sp.
652	Lachnospira_sp.
653	Lachnotalea_sp.
654	Lapillicoccus_sp.
655	Larkinella_insperata
656	Larkinella_rosea
657	Latilactobacillus_sp.
658	Lautropia_sp.
659	Lawsonella_sp.
660	Lebetimonas_sp.
661	Leeia_oryzae
662	Legionella_adelaidensis
663	Legionella_beliardensis
664	Legionella_birminghamensis
665	Legionella_lansingensis
666	Legionella_nautarum
667	Legionella_pittsburghensis
668	Legionella_pneumophila
669	Legionella_quinlivanii
670	Legionella_sp.
671	Lechevalieria_sp.
672	Leifsonia_sp.
673	Lentibacillus_sp.
674	Lentimicrobium_sp.
675	Lentimonas_sp.
676	Leptolinea_sp.

677	Leptospira_sp.
678	Leptothrix_sp.
679	Leucobacter_exalbidus
680	Leucobacter_chromiiresistens
681	Leucobacter_sp.
682	Leuconostoc_gelidum
683	Leuconostoc_lactis
684	Limibaculum_sp.
685	Limnobacter_sp.
686	Limnochorda_sp.
687	Limnothrix_sp.
688	Limosilactobacillus_sp.
689	Litorilinea_sp.
690	Litorilinea_sp.
691	Longilinea_sp.
692	Longimicrobium_sp.
693	Longispora_fulva
694	Longispora_sp.
695	Longivirga_sp.
696	Luedemannella_flava
697	Luedemannella_sp.
698	Luteibacter_anthropi
699	Luteibaculum_sp.
700	Luteimonas_sp.
701	Luteitalea_pratensis
702	Luteitalea_sp.
703	Luteolibacter_sp.
704	Lutispora_sp.
705	Lysinibacillus_alkaliphilus
706	Lysinibacillus_contaminans
707	Lysinibacillus_fusiformis
708	Lysinibacillus_sp.
709	Lysinimicrobium_sp.
710	Lysobacter_dokdonensis
711	Lysobacter_sp.
712	Magnetococcus_sp.
713	Magnetospirillum_sp.
714	Mahella_sp.
715	Marinagarivorans_sp.
716	Marininema_halotolerans
717	Marininema_mesophilum
718	Marinomonas_sp.
719	Marisediminicola_sp.

720	Marixanthomonas_sp.
721	Marmoricola_bigeumensis
722	Marmoricola_sp.
723	Marvinbryantia_sp.
724	Massilia_sp.
725	Massilia_timonae
726	Megasphaera_sp.
727	Mechercharimyces_mesophilus
728	Melghirimyces_sp.
729	Melghirimyces_thermohalophilus
730	Mesorhizobium_sp.
731	Mesorhizobium_temperatum
732	Methylobacter_sp.
733	Methylobacterium-Methylorubrum_aquaticum
734	Methylobacterium-Methylorubrum_organophilum
735	Methylobacterium-Methylorubrum_oxalidis
736	Methylobacterium-Methylorubrum_soli
737	Methylobacterium-Methylorubrum_sp.
738	Methylocaldum_marinum
739	Methylocaldum_szegediense
740	Methylocaldum_tepidum
741	Methylocella_sp.
742	Methylocystis_sp.
743	Methylomarinovum_caldicuralii
744	Methylomicrobium_sp.
745	Methyloprofundus_sp.
746	Methylorosula_sp.
747	Methylotenera_sp.
748	Microbacterium_sp.
749	Microbispora_rosea
750	Micrococcus_sp.
751	Microlunatus_sp.
752	Micromonospora_coerulea
753	Micromonospora_echinospora
754	Micromonospora_chaiyaphumensis
755	Micromonospora_maritima
756	Micromonospora_narathiwatensis
757	Micromonospora_siamensis
758	Micromonospora_sp.
759	Micromonospora_viridifaciens
760	Micropruina_sp.
761	Microvirga_aerilata
762	Microvirga_aerophila

763	Microvirga_sp.
764	Microvirgula_aerodenitrificans
765	Minicystis_rosea
766	Minicystis_sp.
767	Mobilitalea_sp.
768	Modestobacter_marinus
769	Modestobacter_multiseptatus
770	Modestobacter_sp.
771	Moheibacter_sediminis
772	Monoglobus_sp.
773	Moorella_sp.
774	Moorella_thermoacetica
775	Moraxella_lincolnii
776	Motilibacter_peucedani
777	Mucilaginibacter_craterilacus
778	Mucilaginibacter_lutimaris
779	Mucilaginibacter_psychrotolerans
780	Mucilaginibacter_sabulilitoris
781	Mucilaginibacter_sp.
782	Mumia_sp.
783	Mycoavidus_cysteinexigens
784	Mycobacterium_asiaticum
785	Mycobacterium_brisbanense
786	Mycobacterium_cosmeticum
787	Mycobacterium_parmense
788	Mycobacterium_petroleophilum
789	Mycobacterium_rhodesiae
790	Mycobacterium_sp.
791	Mycoplasma_indiense
792	Mycoplasma_sp.
793	Myroides_sp.
794	Myxococcus_sp.
795	Nakamurella_endophytica
796	Nakamurella_flavida
797	Nakamurella_multipartita
798	Nakamurella_sp.
799	Nannocystis_exedens
800	Nannocystis_sp.
801	Natranaerovirga_pectinivora
802	Natronincola_sp.
803	Negadavirga_shengliensis
804	Neisseria_sp.
805	Neochlamydia_hartmannellae

806	Neochlamydia_sp.
807	Niastella_gongjuensis
808	Niastella_sp.
809	Nibribacter_sp.
810	Nitratireductor_sp.
811	Nitriliruptor_alkaliphilus
812	Nitrobacter_vulgaris
813	Nitrobacter_winogradskyi
814	Nitrococcus_sp.
815	Nitrolancea_hollandica
816	Nitrolancea_sp.
817	Nitrosomonas_communis
818	Nitrosomonas_sp.
819	Nitrosospira_sp.
820	Nitrospira_sp.
821	Nitrospirillum_amazonense
822	Nitrospirillum_sp.
823	Nocardia_africana
824	Nocardia_beijingensis
825	Nocardia_carnea
826	Nocardia_gamkensis
827	Nocardia_jiangxiensis
828	Nocardia_jinanensis
829	Nocardia_nova
830	Nocardia_otitidiscaviarum
831	Nocardia_paucivorans
832	Nocardia_sp.
833	Nocardia_uniformis
834	Nocardia_vinacea
835	Nocardioides_aquaticus
836	Nocardioides_humi
837	Nocardioides_koreensis
838	Nocardioides_litorisoli
839	Nocardioides_luteus
840	Nocardioides_pelophilus
841	Nocardioides_plantarum
842	Nocardioides_sp.
843	Nocardioides_szechwanensis
844	Nocardioides_ungokensis
845	Nocardiopsis_sp.
846	Nodosilinea_PCC-7104_sp.
847	Nodularia_PCC-9350_sp.
848	Nonomuraea_flavida

849	Nonomuraea_gerenzanensis
850	Nonomuraea_pusilla
851	Nonomuraea_recticatena
852	Nonomuraea_sp.
853	Nordella_sp.
854	Nostoc_PCC-7107_sp.
855	Novibacillus_sp.
856	Novibacillus_thermophilus
857	Noviherbaspirillum_canariense
858	Noviherbaspirillum_malthae
859	Noviherbaspirillum_psychrotolerans
860	Noviherbaspirillum_sp.
861	Novosphingobium_nitrogenifigens
862	Novosphingobium_sp.
863	Nubsella_sp.
864	Occallatibacter_sp.
865	Oceanicella_sp.
866	Oceanobacillus_endoradicis
867	Oceanobacillus_iheyensis
868	Oceanobacillus_indicireducens
869	Oceanobacillus_limi
870	Oceanobacillus_sp.
871	Ohtaekwangia_sp.
872	Ochrobactrum_anthropi
873	Oikopleura_sp.
874	Oligoflexus_sp.
875	Oligoflexus_tunisiensis
876	Oligotropha_carboxidovorans
877	Olivibacter_soli
878	Olsenella_sp.
879	Opitutus_sp.
880	Orenia_marismortui
881	Ornithinibacillus_contaminans
882	Ornithinibacillus_sp.
883	Ornithinibacter_sp.
884	Oryzihumus_sp.
885	Oryzihumus_terrae
886	Oscillibacter_sp.
887	Oscillochloris_sp.
888	Oscillospira_sp.
889	Oxalobacter_sp.
890	Oxalophagus_sp.
891	Oxobacter_pfennigii

892	Oxobacter_sp.
893	Paenarthrobacter_sp.
894	Paenibacillus_agarexedens
895	Paenibacillus_agaridevorans
896	Paenibacillus_alkaliterrae
897	Paenibacillus_antarcticus
898	Paenibacillus_barcinonensis
899	Paenibacillus_barengoltzii
900	Paenibacillus_bovis
901	Paenibacillus_castaneae
902	Paenibacillus_cavernae
903	Paenibacillus_contaminans
904	Paenibacillus_cookii
905	Paenibacillus_crassostreae
906	Paenibacillus_cucumis
907	Paenibacillus_daejeonensis
908	Paenibacillus_dendritiformis
909	Paenibacillus_doosanensis
910	Paenibacillus_edaphicus
911	Paenibacillus_ehimensis
912	Paenibacillus_eucommiae
913	Paenibacillus_filicis
914	Paenibacillus_gansuensis
915	Paenibacillus_ginsengarvi
916	Paenibacillus_glacialis
917	Paenibacillus_granivorans
918	Paenibacillus_hodogayensis
919	Paenibacillus_chartarius
920	Paenibacillus_chitinolyticus
921	Paenibacillus_ihbetae
922	Paenibacillus_kobensis
923	Paenibacillus_koleovorans
924	Paenibacillus_konkukensis
925	Paenibacillus_larvae
926	Paenibacillus_lautus
927	Paenibacillus_liaoningensis
928	Paenibacillus_macerans
929	Paenibacillus_marinisediminis
930	Paenibacillus_montanisoli
931	Paenibacillus_motobuensis
932	Paenibacillus_mucilaginosus
933	Paenibacillus_panacisoli
934	Paenibacillus_pectinilyticus

935	Paenibacillus_periandrae
936	Paenibacillus_phoenicis
937	Paenibacillus_phyllosphaerae
938	Paenibacillus_pinihumi
939	Paenibacillus_polymyxa
940	Paenibacillus_populi
941	Paenibacillus_profundus
942	Paenibacillus_prosopidis
943	Paenibacillus_quercus
944	Paenibacillus_rhizoryzae
945	Paenibacillus_rigui
946	Paenibacillus_sediminis
947	Paenibacillus_segetis
948	Paenibacillus_sepulcri
949	Paenibacillus_sophorae
950	Paenibacillus_sp.
951	Paenibacillus_stellifer
952	Paenibacillus_tarimensis
953	Paenibacillus_terreus
954	Paenibacillus_terrigena
955	Paenibacillus_thermoaerophilus
956	Paenibacillus_turicensis
957	Paenibacillus_validus
958	Paenibacillus_xylanexedens
959	Paenibacillus_xylanilyticus
960	Paenisporosarcina_sp.
961	Pajaroellobacter_sp.
962	Paludibacter_sp.
963	Paludibacterium_sp.
964	Paludibaculum_sp.
965	Paludicola_sp.
966	Paludisphaera_borealis
967	Paludisphaera_sp.
968	Panacagrimonas_sp.
969	Papillibacter_sp.
970	Paraclostridium_sp.
971	Paracoccus_koreensis
972	Paracoccus_solventivorans
973	Paracoccus_sp.
974	Parafilimonas_sp.
975	Parafrigoribacterium_sp.
976	Parachlamydia_acanthamoebae
977	Paramaledivibacter_sp.

978	Parapusillimonas_sp.
979	Parasediminibacterium_sp.
980	Parasegetibacter_sp.
981	Parasegetibacter_terrae
982	Parvibaculum_sp.
983	Parviterribacter_kavangonensis
984	Parviterribacter_multiflagellatus
985	Parviterribacter_sp.
986	Pasteuria_hartismeri
987	Pasteuria_penetrans
988	Pasteuria_sp.
989	Patulibacter_sp.
990	Paucimonas_sp.
991	Pedobacter_daechungensis
992	Pedobacter_ginsengiterrae
993	Pedobacter_glucosidilyticus
994	Pedobacter_lotistagni
995	Pedobacter_pituitosus
996	Pedobacter_sp.
997	Pedococcus-Phycicoccus_sp.
998	Pedomicrobium_manganicum
999	Pedomicrobium_sp.
1000	Pedosphaera_parvula
1001	Pedosphaera_sp.
1002	Pelosinus_fermentans
1003	Pelosinus_sp.
1004	Pelospora_sp.
1005	Pelotomaculum_schinkii
1006	Pelotomaculum_sp.
1007	Peptococcus_sp.
1008	Peptoniphilus_sp.
1009	Peredibacter_sp.
1010	Phaeodactylibacter_sp.
1011	Phaselicystis_flava
1012	Phaselicystis_sp.
1013	Phenylobacterium_hankyongense
1014	Phenylobacterium_sp.
1015	Phormidesmis_ANT.L52.6_sp.
1016	Phreatobacter_sp.
1017	Phycisphaera_sp.
1018	Phyllobacterium_trifolii
1019	Phytohabitans_sp.
1020	Phytomonospora_cypria

1021	Pilimelia_columellifera
1022	Pirellula_sp.
1023	Piscinibacter_sp.
1024	Planctomicrobium_sp.
1025	Planifilum_composti
1026	Planifilum_fimeticola
1027	Planifilum_sp.
1028	Planococcus_sp.
1029	Planomonospora_parontospora
1030	Planomonospora_sp.
1031	Planosporangium_flavigriseum
1032	Plantactinospora_mayteni
1033	Plesiomonas_sp.
1034	Polaromonas_sp.
1035	Polyangium_fumosum
1036	Polyangium_sp.
1037	Polyangium_spumosum
1038	Polycyclovorans_sp.
1039	Polymorphobacter_sp.
1040	Polymorphospora_rubra
1041	Pontibacter_jeungdoensis
1042	Pontibacter_populi
1043	Pontibacter_ruber
1044	Pontibacter_sp.
1045	Porphyromonas_sp.
1046	Povalibacter_uvarum
1047	Prauserella_marina
1048	Prevotella_intermedia
1049	Procabacter_sp.
1050	Promicromonospora_sukumoe
1051	Promicromonospora_umidemergens
1052	Propionicimonas_sp.
1053	Prosthecobacter_sp.
1054	Prosthecomicrobium_sp.
1055	Proteiniborus_sp.
1056	Proteiniclasticum_sp.
1057	Proteus_mirabilis
1058	Providencia_vermicola
1059	Pseudactinotalea_sp.
1060	Pseudaminobacter_sp.
1061	Pseudobacteriovorax_sp.
1062	Pseudobacteroides_sp.
1063	Pseudoclavibacter_terrae

1064	Pseudoclostridium_sp.
1065	Pseudoclostridium_thermosuccinogenes
1066	Pseudofulvimonas_sp.
1067	Pseudogracilibacillus_auburnensis
1068	Pseudogracilibacillus_sp.
1069	Pseudochrobactrum_kiredjianiae
1070	Pseudolabrys_sp.
1071	Pseudolabrys_taiwanensis
1072	Pseudomonas_composti
1073	Pseudomonas_fluorescens
1074	Pseudomonas_mandelii
1075	Pseudomonas_putida
1076	Pseudomonas_sp.
1077	Pseudomonas_veronii
1078	Pseudomonas_xanthomarina
1079	Pseudonocardia_halophobica
1080	Pseudonocardia_hydrocarbonoxydans
1081	Pseudonocardia_kunmingensis
1082	Pseudonocardia_seranimata
1083	Pseudonocardia_sp.
1084	Pseudonocardia_yunnanensis
1085	Pseudorhodobacter_sp.
1086	Pseudoxanthomonas_gei
1087	Pseudoxanthomonas_putridarboris
1088	Pseudoxanthomonas_sp.
1089	Pseudoxanthomonas_yeongjuensis
1090	Psychrobacillus_insolitus
1091	Psychrobacillus_psychrodurans
1092	Psychrobacillus_sp.
1093	Psychroglaciecola_arctica
1094	Psychroglaciecola_sp.
1095	Psychromonas_sp.
1096	Puia_sp.
1097	Pullulanibacillus_camelliae
1098	Pusillimonas_sp.
1099	Qipengyuania_sp.
1100	Quadrisphaera_sp.
1101	Rahnella_sp.
1102	Ramlibacter_henchirensis
1103	Reyranella_graminifolii
1104	Reyranella_massiliensis
1105	Reyranella_soli
1106	Reyranella_sp.

1107	Rhabdanaerobium_thermarum
1108	Rhizobacter_fulvus
1109	Rhizobacter_sp.
1110	Rhizocola_sp.
1111	Rhizorhapis_sp.
1112	Rhodanobacter_fulvus
1113	Rhodanobacter_sp.
1114	Rhodobaculum_sp.
1115	Rhodoblastus_sp.
1116	Rhodococcus_canchipurensis
1117	Rhodococcus_erythropolis
1118	Rhodococcus_jostii
1119	Rhodococcus_sp.
1120	Rhodococcus_tukisamuensis
1121	Rhodococcus_wratislaviensis
1122	Rhodocytophaga_sp.
1123	Rhodoferax_koreense
1124	Rhodoferax_sp.
1125	Rhodomicrobium_sp.
1126	Rhodopila_globiformis
1127	Rhodopila_sp.
1128	Rhodopirellula_sp.
1129	Rhodoplanes_sp.
1130	Rhodopseudomonas_sp.
1131	Rhodovarius_sp.
1132	Rhodovastum_sp.
1133	Rickettsia_sp.
1134	Rickettsiella_sp.
1135	Risungbinella_sp.
1136	Robinsoniella_sp.
1137	Romboutsia_sp.
1138	Roseburia_sp.
1139	Roseiarcus_sp.
1140	Roseicyclus_sp.
1141	Roseimicrobium_sp.
1142	Roseisolibacter_agri
1143	Roseisolibacter_sp.
1144	Rosenbergiella_sp.
1145	Roseomonas_aquatica
1146	Roseomonas_frigidaquae
1147	Roseomonas_sp.
1148	Roseomonas_terricola
1149	Rothia_amarae

1150	Rothia_sp.
1151	Rubellimicrobium_aerolatum
1152	Rubellimicrobium_sp.
1153	Rubritepida_sp.
1154	Rubrobacter_sp.
1155	Rufibacter_sp.
1156	Rugosimonospora_acidiphila
1157	Ruminiclostridium_cellulolyticum
1158	Ruminiclostridium_hungatei
1159	Ruminiclostridium_sp.
1160	Ruminiclostridium_sufflavum
1161	Ruminococcus_sp.
1162	Rummeliibacillus_sp.
1163	Rummeliibacillus_stabekisii
1164	Rurimicrobium_sp.
1165	Saccharibacillus_deserti
1166	Saccharomonospora_viridis
1167	Saccharopolyspora_cavernae
1168	Saccharopolyspora_erythraea
1169	Saccharopolyspora_gloriosae
1170	Saccharopolyspora_hirsuta
1171	Saccharopolyspora_rectivirgula
1172	Saccharopolyspora_sp.
1173	Saccharothrix_sp.
1174	Salicola_sp.
1175	Salinispora_sp.
1176	Salipaludibacillus_sp.
1177	Saliterribacillus_sp.
1178	Salmonella_enterica
1179	Salmonella_sp.
1180	Sandaracinus_amylolyticus
1181	Sandaracinus_sp.
1182	Sanguibacter-Flavimobilis_suarezii
1183	Sarcina_maxima
1184	Sarcina_sp.
1185	Scytonema_PCC-7110_sp.
1186	Sedimentibacter_hongkongensis
1187	Sedimentibacter_sp.
1188	Sediminibacterium_goheungense
1189	Sediminibacterium_sp.
1190	Segetibacter_koreensis
1191	Segetibacter_sp.
1192	Seinonella_peptonophila

1193	Seinonella_sp.
1194	Serinibacter_sp.
1195	Serratia_plymuthica
1196	Serratia_sp.
1197	Shimazuella_kribbensis
1198	Shimazuella_sp.
1199	Schlegelella_thermodepolymerans
1200	Schlesneria_sp.
1201	Schumannella_sp.
1202	Siccirubricoccus_deserti
1203	Sideroxydans_paludicola
1204	Sideroxydans_sp.
1205	Silvanigrella_sp.
1206	Simkania_negevensis
1207	Singulisphaera_sp.
1208	Sinibacillus_soli
1209	Sinibacillus_sp.
1210	Skermanella_rubra
1211	Skermanella_sp.
1212	Smaragdicoccus_sp.
1213	Solibacillus_silvestris
1214	Solibacillus_sp.
1215	Solimonas_sp.
1216	Solirubrobacter_sp.
1217	Solitalea_canadensis
1218	Solitalea_sp.
1219	Solobacterium_sp.
1220	Sorangium_cellulosum
1221	Sorangium_sp.
1222	Sphaerimonospora_sp.
1223	Sphaerisporangium_flaviroseum
1224	Sphaerisporangium_sp.
1225	Sphaerobacter_sp.
1226	Sphaerobacter_thermophilus
1227	Sphingoaurantiacus_sp.
1228	Sphingobacterium_sp.
1229	Sphingobium_rhizovicinum
1230	Sphingobium_sp.
1231	Sphingomonas_glacialis
1232	Sphingomonas_rhizophila
1233	Sphingomonas_sediminicola
1234	Sphingomonas_soli
1235	Sphingomonas_sp.

1236	Sphingopyxis_alaskensis
1237	Sphingorhabdus_sp.
1238	Sphingorhabdus_wooponensis
1239	Spirochaeta_2_sp.
1240	Spirochaeta_sp.
1241	Spirosoma_endophyticum
1242	Spirosoma_litoris
1243	Spirosoma_panaciterrae
1244	Spirosoma_sp.
1245	Spongiimonas_sp.
1246	Sporacetigenium_sp.
1247	Sporanaerobacter_acetigenes
1248	Sporanaerobacter_sp.
1249	Sporichthya_sp.
1250	Sporocytophaga_myxococcoides
1251	Sporocytophaga_sp.
1252	Sporolactobacillus_sp.
1253	Sporomusa_acidovorans
1254	Sporomusa_aerivorans
1255	Sporomusa_malonica
1256	Sporomusa_sp.
1257	Sporomusa_sphaeroides
1258	Sporosarcina_koreensis
1259	Sporosarcina_pasteurii
1260	Sporosarcina_psychrophila
1261	Sporosarcina_sp.
1262	Stackebrandtia_endophytica
1263	Stackebrandtia_soli
1264	Stackebrandtia_sp.
1265	Staphylococcus_sp.
1266	Staphylococcus_vitulinus
1267	Stenotrophobacter_sp.
1268	Stenotrophobacter_terrae
1269	Stenotrophomonas_daejeonensis
1270	Stenotrophomonas_maltophilia
1271	Stenotrophomonas_sp.
1272	Steroidobacter_sp.
1273	Sterolibacterium_sp.
1274	Stigmatella_erecta
1275	Stigmatella_sp.
1276	Streptacidiphilus_sp.
1277	Streptococcus_salivarius
1278	Streptococcus_sp.

1279	Streptomyces_aculeolatus
1280	Streptomyces_albiaxialis
1281	Streptomyces_atratus
1282	Streptomyces_aureus
1283	Streptomyces_bluensis
1284	Streptomyces_cattleya
1285	Streptomyces_cinnamonensis
1286	Streptomyces_citricolor
1287	Streptomyces_coeruleorubidus
1288	Streptomyces_echinoruber
1289	Streptomyces_exfoliatus
1290	Streptomyces_griseoplanus
1291	Streptomyces_griseoruber
1292	Streptomyces_griseus
1293	Streptomyces_haliclonae
1294	Streptomyces_hiroshimensis
1295	Streptomyces_humidus
1296	Streptomyces_palmae
1297	Streptomyces_phaeoluteigriseus
1298	Streptomyces_platensis
1299	Streptomyces_pulveraceus
1300	Streptomyces_puniceus
1301	Streptomyces_scabiei
1302	Streptomyces_sp.
1303	Streptomyces_specialis
1304	Streptomyces_spiralis
1305	Streptomyces_sulfonofaciens
1306	Streptomyces_thermogriseus
1307	Streptomyces_tritici
1308	Streptomyces_vastus
1309	Streptomyces_violaceoruber
1310	Streptosporangium_longisporum
1311	Subtercola_vilae
1312	Sulfuriferula_sp.
1313	Sulfurifustis_sp.
1314	Sulfurimonas_sp.
1315	Sulfuritalea_hydrogenivorans
1316	Sumerlaea_sp.
1317	Sunxiuqinia_sp.
1318	Suttonella_sp.
1319	Symbiobacterium_sp.
1320	Symbiobacterium_thermophilum
1321	Syntrophaceticus_sp.

1322	Syntrophobacter_fumaroxidans
1323	Syntrophobacter_sp.
1324	Syntrophomonas_sp.
1325	Syntrophorhabdus_sp.
1326	Syntrophotalea_sp.
1327	Syntrophothermus_sp.
1328	Tahibacter_sp.
1329	Taibaiella_koreensis
1330	Taibaiella_sp.
1331	Tardiphaga_sp.
1332	Telmatocola_sp.
1333	Telmatospirillum_sp.
1334	Tepidanaerobacter_acetatoxydans
1335	Tepidanaerobacter_sp.
1336	Tepidanaerobacter_syntrophicus
1337	Tepidibacter_sp.
1338	Tepidimicrobium_ferriphilum
1339	Tepidimicrobium_sp.
1340	Tepidimicrobium_xylanilyticum
1341	Tepidiphilus_sp.
1342	Tepidisphaera_sp.
1343	Terrabacter_sp.
1344	Terribacillus_goriensis
1345	Terribacillus_sp.
1346	Terrimicrobium_sp.
1347	Terrimonas_arctica
1348	Terrimonas_sp.
1349	Terrisporobacter_sp.
1350	Tetrasphaera_sp.
1351	Thalassobacillus_sp.
1352	Thermacetogenium_sp.
1353	Thermaerobacter_subterraneus
1354	Thermanaeromonas_sp.
1355	Thermasporomyces_composti
1356	Thermicanus_aegyptius
1357	Thermincola_sp.
1358	Thermoactinomyces_daqus
1359	Thermoactinomyces_intermedius
1360	Thermoactinomyces_khenchelensis
1361	Thermoactinomyces_sp.
1362	Thermoactinomyces_vulgaris
1363	Thermoanaerobacterium_sp.
1364	Thermobacillus_composti

1365	Thermobacillus_sp.
1366	Thermobacillus_xylanilyticus
1367	Thermobaculum_sp.
1368	Thermobifida_fusca
1369	Thermobispora_bispora
1370	Thermocatellispora_sp.
1371	Thermoclostridium_sp.
1372	Thermoflavimicrobium_sp.
1373	Thermomonas_sp.
1374	Thermomonospora_curvata
1375	Thermomonospora_sp.
1376	Thermopolyspora_sp.
1377	Thermorudis_peleae
1378	Thermosediminibacter_oceani
1379	Thermosporothrix_sp.
1380	Thermostaphylospora_chromogena
1381	Thermovenabulum_sp.
1382	Thermus_scotoductus
1383	Thioalkalispira-Sulfurivermis_sp.
1384	Thiobacillus_sp.
1385	Thiothrix_sp.
1386	Tissierella_carlieri
1387	Tissierella_creatinini
1388	Tissierella_sp.
1389	Tistlia_sp.
1390	Tolypothrix_sp.
1391	Treponema_sp.
1392	Trichormus_HINDAK_2001-4_sp.
1393	Truepera_sp.
1394	Tuberibacillus_calidus
1395	Tuberibacillus_sp.
1396	Tumebacillus_algifaecis
1397	Tumebacillus_flagellatus
1398	Tumebacillus_ginsengisoli
1399	Tumebacillus_luteolus
1400	Tumebacillus_sp.
1401	Tundrisphaera_lichenicola
1402	Tundrisphaera_sp.
1403	Turicella_sp.
1404	Turicibacter_sp.
1405	Turneriella_sp.
1406	Tychonema_CCAP_1459-11B_sp.
1407	Tyzzerella_sp.

1409	Uliginosibacterium_sp.
1410	Umezawaea_endophytica
1411	Umezawaea_tangerina
1412	Undibacterium_sp.
1413	Ureibacillus_sp.
1414	Ureibacillus_thermosphaericus
1415	Vagococcus_sp.
1416	Vampirovibrio_chlorellavorus
1417	Vampirovibrio_sp.
1418	Variovorax_paradoxus
1419	Variovorax_sp.
1420	Veillonella_sp.
1421	Verminephrobacter_aporrectodeae
1422	Verrucomicrobium_sp.
1423	Verrucosispora_sonchi
1424	Verrucosispora_sp.
1425	Verticiella_sp.
1426	Vibrio_sp.
1427	Vicinamibacter_sp.
1428	Virgibacillus_sp.
1429	Virgisporangium_myanmarense
1430	Virgisporangium_sp.
1431	Vitellibacter_sp.
1432	Vogesella_alkaliphila
1433	Vogesella_sp.
1434	Vulcanibacillus_sp.
1435	Vulgatibacter_incomptus
1436	Vulgatibacter_sp.
1437	Wangella_sp.
1438	Williamsia_limnetica
1439	Woeseia_sp.
1440	Wolbachia_sp.
1441	Xylanibacillus_composti
1442	Xylanibacillus_sp.
1443	Yersinia_frederiksenii
1444	Yersinia_pestis
1445	Yersinia_ruckeri
1446	Zavarzinella_formosa
1447	Zavarzinella_sp.
1448	Zhihengliuella_alba
